# Characterization of head movement patterns in patients with bilateral and unilateral vestibulopathy during functional mobility tasks

**DOI:** 10.3389/fnins.2026.1731221

**Published:** 2026-02-11

**Authors:** Gautier Grouvel, Julie Corre, Maurizio Ranieri, Samuel Cavuscens, Anissa Boutabla, Jean-François Cugnot, Raymond van de Berg, Nils Guinand, Stéphane Armand, Angélica Pérez Fornos

**Affiliations:** 1 Division of Otorhinolaryngology Head and Neck Surgery, Geneva University Hospitals and University of Geneva, Geneva, Switzerland; 2 Kinesiology Laboratory, Geneva University Hospitals and University of Geneva, Geneva, Switzerland; 3 Centre of Research on Skeletal Muscle and Movement, Geneva University Hospitals and University of Geneva, Geneva, Switzerland; 4 Division of Neurorehabilitation, Clinical Neurosciences Department, Geneva University Hospitals, Geneva, Switzerland; 5 Division of Balance Disorders, Department of Otorhinolaryngology and Head and Neck Surgery, Maastricht University Medical Center+, Maastricht, Netherlands

**Keywords:** angular acceleration, angular velocity, functional mobility tasks, head movements, inertial sensors, vestibulogram, vestibulopathy

## Abstract

**Introduction:**

Bilateral and unilateral vestibulopathies mainly cause chronic imbalance/unsteadiness and oscillopsia, significantly impacting quality of life. Traditional clinical tests fail to assess functional impact on daily activities. This study aimed to characterize head movement patterns in bilateral vestibulopathy (BV) and unilateral vestibulopathy (UV) patients during functional mobility tasks in a semi-standardized environment. This study could provide useful information for rehabilitation and optimization of vestibular implant stimulation.

**Methods:**

Fifty-nine participants (19 BV, 20 UV, 20 healthy subjects) performed 10 functional mobility tasks, and a subtask extracted during analysis, while wearing inertial measurement units that recorded head angular velocities. Angular accelerations were derived from these. Vector norms and mode values of distribution histograms were calculated for both variables. Statistical analyses were performed using linear mixed-effects models to compare head movement parameters (angular velocity and angular acceleration) between groups (healthy subjects vs. patients) and tasks (walk vs. other tasks), including group × task interactions. Correlation analyses were also performed to compare the objective values with the patients’ perception of task difficulty.

**Results:**

Vestibulopathy patients demonstrated significantly reduced head movements compared to healthy subjects. BV patients showed the most restrictive patterns (angular velocities estimated between 8 and 12 deg/s; accelerations between 85 and 150 deg/s^2^, with statistically significant main effects of group and task and specific significant group × task interactions). UV patients exhibited intermediate values with greater variability. Healthy subjects displayed task-specific adaptations and higher movement ranges (Q1-Q3 area: 5.97 vs. 3.58 vs. 2.69 deg^2^/s^3^ (angular velocity x angular acceleration) for healthy, UV, and BV groups respectively).

**Conclusion:**

Vestibulopathy leads to compensatory head stiffening strategies specific to certain tasks, with BV patients exhibiting the most pronounced limitations. These findings may suggest that patients adopt cautious motor behaviors during their functional mobility activities. This ecological assessment provides essential parameters for optimizing vestibular rehabilitation protocols and defining realistic technical requirements for vestibular implants.

## 1 Introduction

The vestibular system plays a crucial role in the perception of head movements (Cullen and Sadeghi, 2008). It detects angular accelerations, predominantly via the semicircular canals, and linear accelerations (including gravity) and tilts, predominantly via the otolith organs. The vestibular system may be damaged, and consequently vestibular function may be unilaterally or bilaterally absent or severely impaired. In both patients with unilateral vestibulopathy (UV) and bilateral vestibulopathy (BV), chronic imbalance/unsteadiness is a cardinal symptom (Lucieer et al., 2020; Karabulut et al., 2023). BV and UV impose a significant burden on patients, leading to a decrease of the quality of life (Lucieer et al., 2020; Guinand et al., 2012; Karabulut et al., 2024). Several diagnostic tests are available to assess peripheral vestibular function, using vestibulo-ocular reflex tests (e.g., calorics, rotatory chair and video-head impulse test) or vestibulo-collic/spinal reflex tests (e.g., cervical vestibular evoked myogenic potentials, posturography). Although these clinical tests have proven to be useful to assess the functional status of semicircular canals and otolithic organs, they do not allow to understand the functional impact of such deficits on the performance of functional mobility tasks, i.e., tasks that closely reproduce real life activities.

Several studies have already analyzed head movements during controlled laboratory environments (i.e., in-hospital) and shown that, to limit disturbances and imbalance, patients with vestibular deficits adopt rigid head stabilization strategies, i.e., head-trunk fixed together. Pozzo et al. (1991) were among the first to demonstrate that subjects with bilateral vestibular deficits were unable to maintain the Frankfurt plane horizontal during walking (Hofmann et al., 2016), reflecting an inability to stabilize the head in space (Pozzo et al., 1991). Mamoto et al. (2002) confirmed these observations, reporting increased head and trunk rotations in these patients. Patients with unilateral and bilateral vestibular deficits adopted rigid head stabilization strategies in the trunk (‘en bloc’), whereas healthy subjects adopted stabilization strategies in space (Mamoto et al., 2002). This was also recently shown in previous work by our research team, showing that UV and BV patients tended to adopt more rigid head stabilization strategies than healthy subjects (Grouvel et al., 2024). Mijovic et al. (2014) were among the first to investigate head movements in UV patients during the performance of functional mobility tasks, using inertial measurement units (IMU). They observed that these patients showed distinct, reduced head movement patterns compared to healthy subjects, although these differences were not correlated with perceived disability (Mijovic et al., 2014). Jabri et al. (2022) extended this approach by analyzing a set of complex motor tasks using 13 IMUs. They also developed a machine-learning algorithm designed to automatically classify vestibular gait abnormalities in UV and BV patients. Their study compared different sensor locations and task types, but the results failed to demonstrate reliable classification of pathology based on data from the sensor placed on the head (Jabri et al., 2022). Investigating these results further, particularly in BV patients, and understanding how these patients control their head movements in their functional mobility activities is crucial (Herssens et al., 2020), not only to better characterize their limitations and disability, but also to evaluate and optimize current and future rehabilitation therapies, such as vestibular physiotherapy or vestibular implants (Guinand et al., 2015; Ramos Macias et al., 2020; Della Santina et al., 2007).

The aim of this study was to characterize head movements, specifically angular velocities and angular accelerations, in BV and UV patients, compared to a healthy subjects (HS) group in a semi-standardized environment reproducing functional mobility tasks representative of daily activities. More specifically, we wanted to identify the strategies adopted by the different groups through the analysis of head movements during the performance of different locomotor tasks, some of which—such as walking on an uneven ground or performing a UTurn—represent particular challenges for BV patients. To this end, we quantified head movement speeds as a function of task-specific requirements, to identify distinctive profiles between groups. Furthermore, this approach could provide useful insights for refining the technical requirements of vestibular implants.

We hypothesized that BV and UV patients have lower head velocities and angular accelerations than the HS group, and that the BV group has lower values than the UV group.

## 2 Methods

This exploratory and observational study aimed to describe head motion patterns associated with bilateral and unilateral vestibulopathy and compare them to control subjects (i.e., those with normal vestibular function).

### 2.1 Participants

Three groups of participants were included in this study: a chronic bilateral vestibulopathy group (BV), a chronic unilateral vestibulopathy group (UV), and a group of healthy subjects (HS). BV patients were recruited according to the Bárány Society diagnostic criteria for bilateral vestibulopathy (Strupp et al., 2017), which define the disorder on the basis of typical symptomatology and bilaterally reduced lateral semicircular canal vestibulo-ocular reflex (VOR) function. All BV participants underwent comprehensive vestibular testing, including six-canal vHIT and caloric testing. For our inclusion criteria, we consistently applied vHIT based lateral semicircular canal thresholds following one of the accepted diagnostic routes and adhering to the intent of the consensus framework. Because no consensus diagnostic criteria currently exist for chronic unilateral vestibulopathy, participants in the UV group were included based on lateral-canal vHIT findings, consistent with unilateral VOR impairment. Inclusion required: a lateral semicircular canal gain < 0.6 on the affected side, a gain > 0.8 on the contralateral side, symptom duration ≥ 3 months, and no evidence of other otologic or neurologic disease. This ensured methodological consistency across groups, as the same lateral-canal-based criteria was used for classifying BV participants. Finally, all HS were screened through a comprehensive structured interview, which confirmed no history of dizziness, vertigo, or imbalance, no hearing loss or otologic disease, no neurologic conditions, no prior exposure to ototoxic medication, no other medical conditions affecting vestibular or balance function. vHIT was not performed in HS, as their asymptomatic status and absence of risk factors were confirmed through detailed history. This approach is consistent with common practice in vestibular research when laboratory testing is not required for control group classification.

All study participants were over 18 years of age and provided their written informed consent. The population studied was identical to that of the previous study described in the published data descriptor (Grouvel et al., 2025a). The study was designed and conducted in accordance with the guidelines of the Declaration of Helsinki and was approved by the Cantonal Commission for Research Ethics of Geneva (BASEC-ID: 2024-02394).

### 2.2 Equipment

Nine IMUs (Physilog6S, MindMaze, Lausanne, Switzerland) sampled at 128 Hz were attached to the participant’s body. No sensor calibration was performed, as recommended by the manufacturer. For this study, we only analyzed the data from the IMU placed on the head. The IMU was attached to the left side of a helmet worn by the participant with a Velcro strap, to minimize measurement errors associated with the positioning of the IMU. It recorded 3-dimensional angular velocity with a range of ± 2000 °/s, and 3-dimensional linear acceleration with a range of ± 16 g during each trial (i.e., task) performed. Data recorded and IMUs properties are presented in more detail in a data descriptor (Grouvel et al., 2025a).

### 2.3 Protocol

Each participant completed a set of 10 functional mobility tasks. The tasks were selected to best represent the limitations and difficulties of patients with bilateral and unilateral vestibulopathies (Mijovic et al., 2014; Jacobson and Newman, 1990; Cohen and Kimball, 2000). All the measurements were carried out during a single session of one hour on a rehabilitation track at the Geneva University Hospitals. This track, which was partially open to the outside environment, provided a semi-standardized setting in which tasks could be reproduced across participants. The acquisitions took place over several months with seasonal variability in weather conditions. The protocol is presented in more detail in another study (Corre et al., 2025), which explains, among other things, why each task is a challenge for BV and UV patients. The tasks performed are shown in Table 1. After completing each task, participants were asked to rate the difficulty of the task as easy, medium, or difficult. An additional task was identified and subsequently included in the analysis, based on data extracted from the Heavy load task, for a total of 11 tasks studied (Table 1).

**Table 1:** List and description of tasks, extracted from Corre et al. (2025).

Tasks	Description
Sorting	The subject sorts plastic crockery from a storage box onto a shelf as quickly as possible according to color and size.
Heavy load	The subject must carry a fully loaded bucket (5 kg) for 10 m, turn around a cone, change hands and return to the starting point (total: 20 m).
Stairs	The subject must climb 8 straight steps and descend 6 spiral steps. If possible, without holding the handrail.
Uneven ground	The subject walks on an unstable cobbled path (15 m).
Tray	The subject must carry two glasses of water on a tray over 12 m without dropping them. The glasses were filled to around 90% of their capacity, with a total weight of approximately 350 g.
Walk	The subject walks in a straight line (12 m) at her/his most comfortable pace.
Wood beam	The subject walks on a 4.5 m wooden beam (15 cm width), turns around and walks back.
Inclined plane	The subject climbs a ramp (25 m – 15°), turns around, and descends it again, the first half of the descent being made with eyes closed.
Picture recognition	Pictures of scenes from daily life are hung on the windows of the room. The subject looks at the pictures (29.7 × 21 cm) while walking (1 round trip: 20 m) and tells the examiner what he/she has seen, giving as much detail as possible. The subject must not stop while walking.
Walk in the dark	Subject walks with welding glasses, which allow vision but greatly reduce light intensity, on level ground for 12 m. The glasses are made of 5 A1 DIN lenses, which allow approximately 5% of light to pass through.
UTurn	The subject makes a U-turn. Task extracted from the “Heavy load” task.

### 2.4 Data processing and analysis

Data processing and analysis were performed using Matlab R2022b (The MathWorks Inc., Natick, MA, United States). Once the IMU data were extracted and imported into Matlab, the gyroscope values, corresponding to angular velocities, were isolated for analysis.

**Figure 1:**
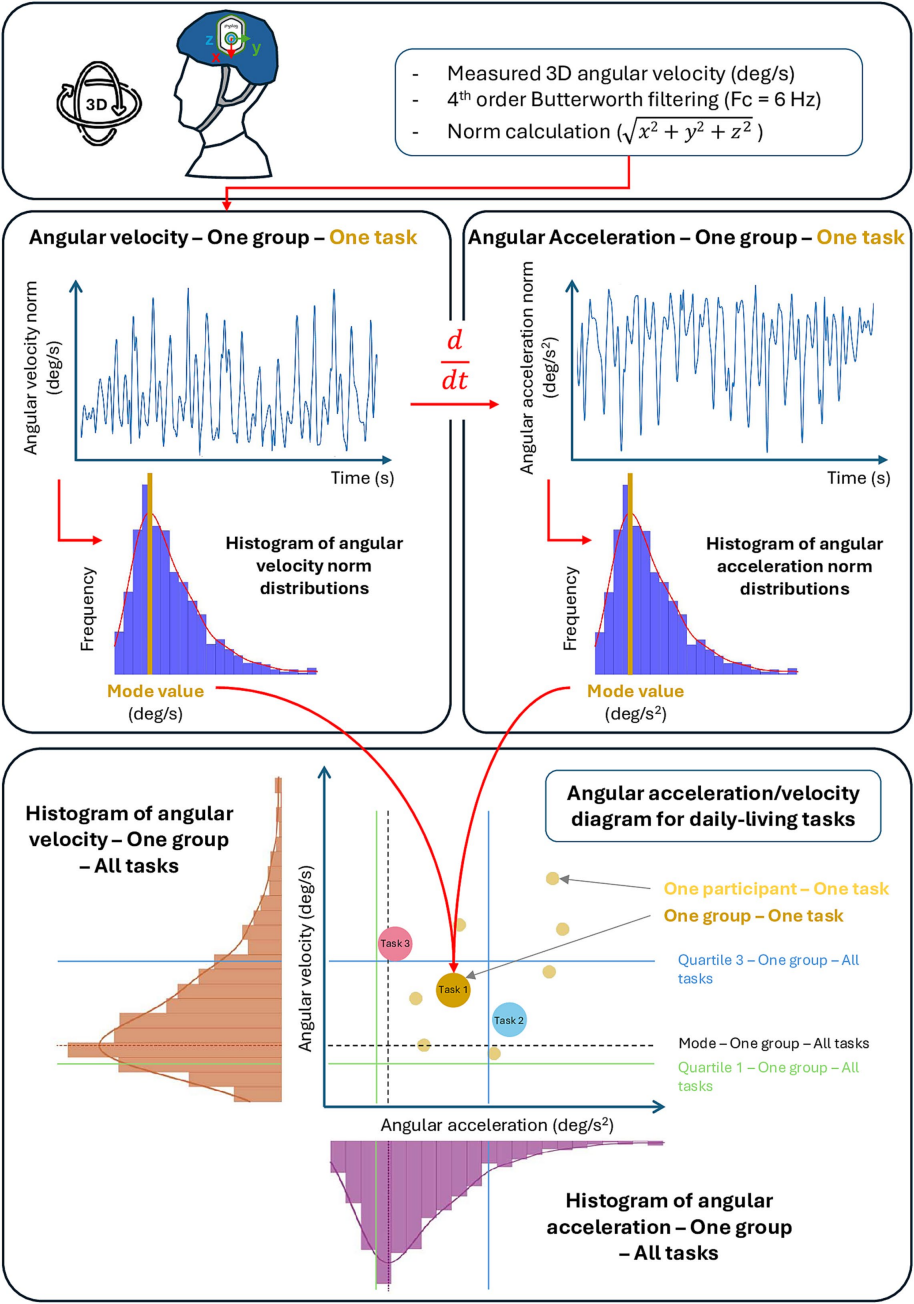
Flow chart of the data processing and analysis.

- Signal processing:
  - The initial and final 5% of each recording were removed to eliminate low quality data, generally associated with the initialization or termination phases of the task (artefacts due to the start-up of the recording or temporary non-compliance with the instructions).
  - Angular velocities were then filtered using a low-pass 4ᵉ order Butterworth filter, with a cut-off frequency set at 6 Hz (Ullrich et al., 2020) (Figure 1).
  - The vector norm of the angular velocities was then calculated (Figure 1) to correct potential alignment errors between the IMU and the anatomical axes of the head, and to represent the overall intensity of the rotational movement, irrespective of its direction.
  - Calculation of angular accelerations: To better characterize head movements during functional mobility tasks, and with the objective to provide potential information for the coding strategies (i.e., transfer function and technical requirements) of future vestibular implants, angular accelerations were also included in the analysis. This parameter corresponds to the variations in rotation speed detected physiologically by the semicircular canals (Angelaki and Cullen, 2008). Angular accelerations were obtained by temporal derivation of each component of the angular velocity (Figure 1).
  - Angular accelerations were also filtered using a low pass 4ᵉ order Butterworth filter, with a cut-off frequency set at 6 Hz, and the vector norm was calculated.
- Extraction of relevant indicators:
  - For each task, distribution histograms (Figure 1) were constructed from the norms of the angular velocities and accelerations. Given the non-normality of the observed distributions, the mode (Figure 1)—corresponding to the most frequent value—was chosen to best represent the most characteristic head movement profile for each task.

The analyses were carried out (a) individually, for each task and each participant, and (b) by task, pooling all the participants within the group. Finally, the histogram of all the data for a group—all the tasks and all the participants—was used to extract the first (Q1) and third (Q3) quartiles in order to quantify intra-group variability and compare the 3 groups with each other (Figure 1).

### 2.5 Statistical analysis

To investigate differences in head movement between groups (healthy subjects vs. patients) and tasks (Walk vs. other tasks), linear mixed-effects models were selected and adjusted to analyze angular velocity and angular acceleration. To provide an adequate model fit, a mean structure (fixed effects) and a covariance structure (random effects) were specified. The mean structure included a group effect (healthy subjects (HS) versus patients: BV and UV), a task effect (Walk versus other tasks), and an interaction effect between group and task. Random effects were used to model inter-individual variability, with a random intercept per participant. For the *j*th observation in participant *i*, in group *g* and task *t*, the response for the different head movement measures (angular velocity: av., and angular acceleration: aa) was modelled as follows:


$$\begin{array}{l} \text{Response}\_av_{\mathrm{ij}}=\alpha +\alpha_{i}+\beta_{1}(\text{Grou}p_{g})+\beta_{2}(\mathrm{Tas}k_{t})+\\ \beta_{3}(\text{Grou}p_{g}\times \mathrm{Tas}k_{t})+\varepsilon_{\mathrm{ij}} \end{array}$$

where *α* is the fixed intercept, α*_i_* is the random intercept for participant *i*, β_1_, β_2_ and β_3_ are the regression coefficients for the main effects and interaction, and ε_ij_ is the measurement error.

Given the repeated nature of the measurements per participant, robust standard errors were calculated using the cluster-robust variance correction method (Huber-White) with clustering by participant. Significance tests were performed using Satterthwaite’s approximation of degrees of freedom, adapted to mixed models. The significance level was set at *p* < 0.05. Healthy subjects (HS) and the walking task (Walk) were defined as reference groups for comparisons. All analyses were performed with R software (version 4.5.1), using the lme4 package for mixed model fitting, lmerTest package for significance testing, and clubSandwich package for robust variance corrections.

## 3 Results

Fifty-nine participants were included in this study: 19 in the BV group, 20 in the UV group, and 20 in the HS group. The participants’ characteristics are presented in Table 2.

**Table 2:** Participants’ characteristics.

Characteristics		Bilateral vestibulopathy (BV) patients (*n* = 19)	Unilateral vestibulopathy (UV) patients (*n* = 20)	Healthy subjects (HS) (*n* = 20)
Sex		11 females	10 females	10 females
Age (years)	Mean (SD)	60.2 (11.6)	59.5 (5.5)	57.9 (5.3)
Height (m)	Mean (SD)	1.69 (0.86)	1.73 (0.10)	1.72 (0.84)
Weight (kg)	Mean (SD)	71.5 (13.7)	74.5 (13.5)	72.1 (13.5)
BMI (kg/m^2^)	Mean (SD)	24.9 (3.5)	24.7 (3.4)	24.3 (4.1)
Affected side		Both sides	11 Right; 9 Left	Not relevant

**Figure 2:**
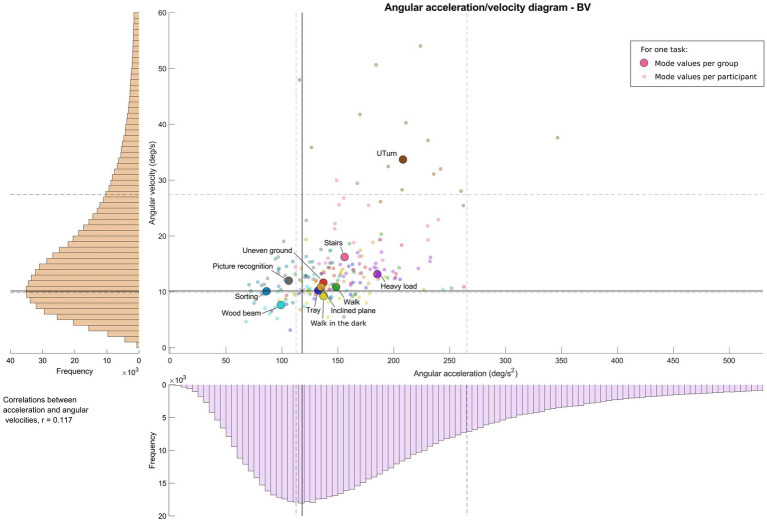
Angular acceleration-velocity diagram for functional mobility tasks for bilateral vestibulopathy patients, “Vestibulogram.” Left and bottom: histogram of distribution for one group and all tasks. Middle: angular acceleration-velocity diagram with wider colored symbols (one task, all participants of the group) and the small symbols (one task, one participant).

**Figure 3:**
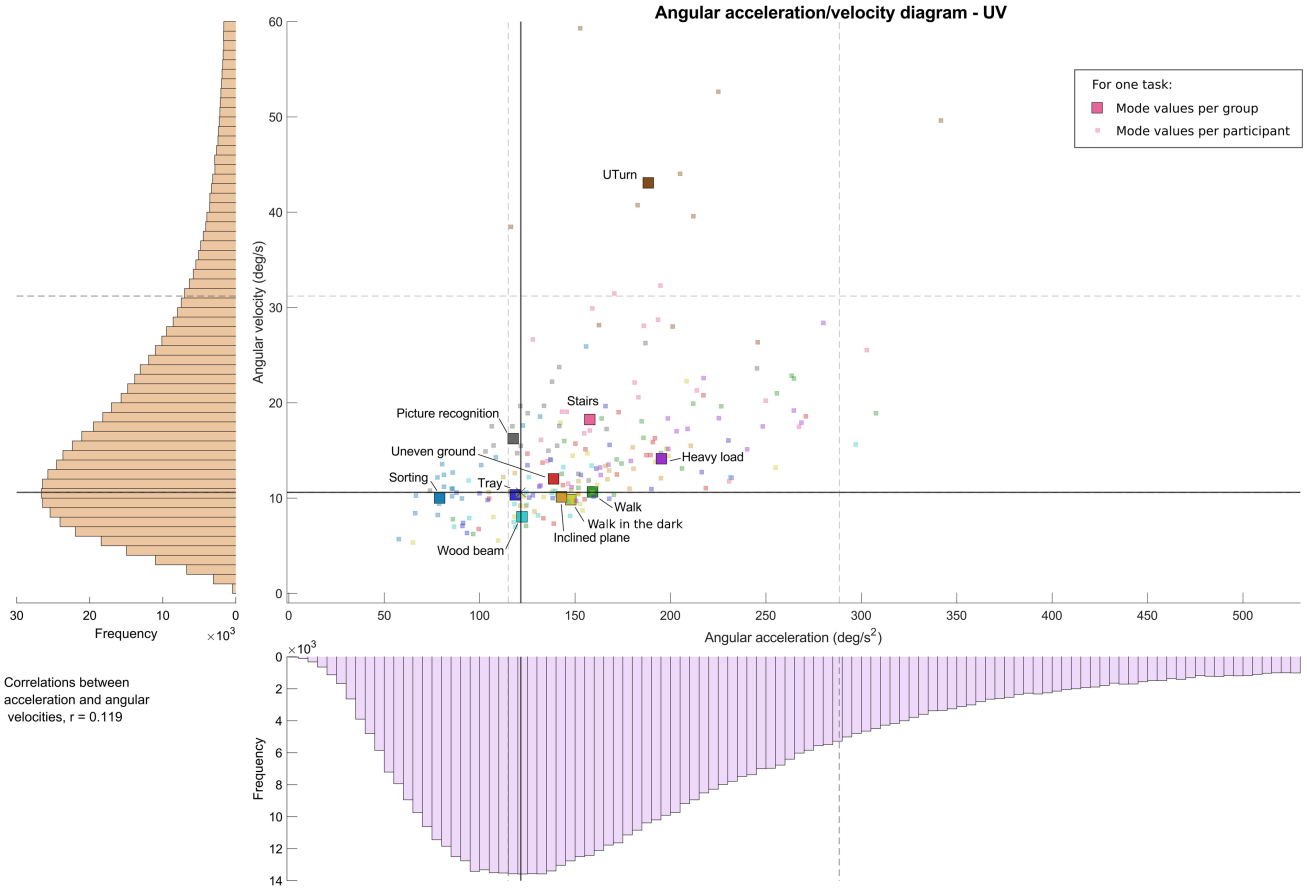
Angular acceleration-velocity diagram for functional mobility tasks for unilateral vestibulopathy patients, “Vestibulogram.” Left and bottom: histogram of distribution for one group and all tasks. Middle: Angular acceleration-velocity diagram with wider colored symbols (one task, all participants of the group) and the small symbols (one task, one participant).

**Figure 4:**
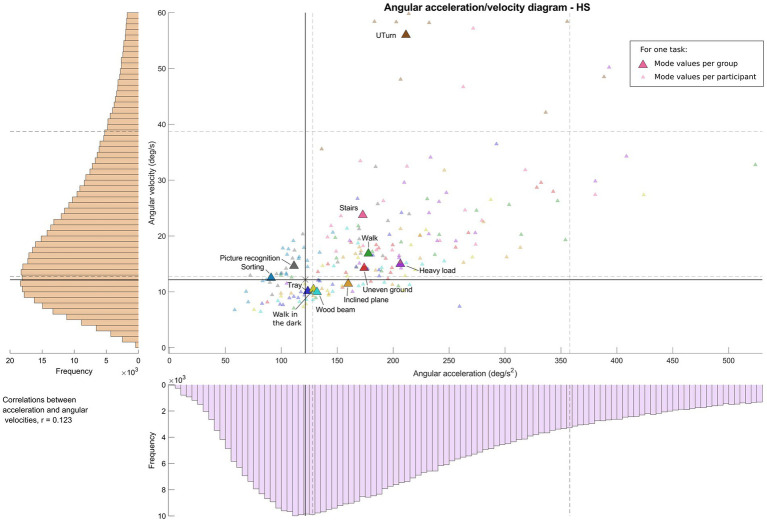
Angular acceleration-velocity diagram for functional mobility tasks for healthy subjects, “Vestibulogram.” Left and bottom: histogram of distribution for one group and all tasks. Middle: Angular acceleration-velocity diagram with wider colored symbols (one task, all participants of the group) and the small symbols (one task, one participant).

Figures 2–4 show, for each task, the values of the angular velocity and angular acceleration modes obtained from all trials for the BV, UV, and HS participant group, respectively. The larger colored symbols (circle: BV; square: UV; triangle: HS) correspond to the mode of the distribution for a given task, calculated for all participants in a group. The smaller symbols represent the individual mode of each participant in the group for the same task. Overall distributions (all tasks and participants) are also shown for both angular velocities and accelerations (vertical and horizontal axes, respectively), as well as the first and third quartiles (Q1 and Q3). Angular velocities and accelerations were poorly correlated in the three groups (BV: r = 0.117; UV: r = 0.119; HS: r = 0.123).

For the BV group (Figure 2), predominant angular velocities were distributed between 8 deg/s and 12 deg/s for the majority of tasks. Angular accelerations ranged between 85 and 150 deg/s^2^. The Stairs and Heavy load tasks exhibited slightly higher values, with 16 deg/s and 155 deg/s^2^, and 13 deg/s and 185 deg/s^2^, respectively. The UTurn task presented the highest angular velocities, reaching values close to 35 deg/s. Except for the UTurn and Wood beam tasks, the majority of tasks and participants are located between the Q1 (10 deg/s, 112 deg/s^2^) and Q3 (27 deg/s, 265 deg/s^2^) quartiles of the overall group distribution. Regarding the group effect (Table 3), compared to references (HS group and Walk task), the BV group showed significantly reduced angular velocities (estimate = −5.62, 95% CI: [−8.99; −2.26], *p* = 0.002, *Δ* = −130.2%) and significantly reduced angular acceleration (estimate = −74.11, 95% CI: [−124.84; −23.38], *p* = 0.005, Δ = −132.8%).

**Table 3:** Linear mixed-effects model results for angular velocity and angular acceleration parameters between groups and during different tasks.

	Angular velocity av (deg/s)			Angular acceleration aa (deg/s^2^)		
Covariate	Estimate	95% CI	*p*-value Δ	Estimate	95% CI	*p*-value *Δ*
(Intercept) HS/Walk	18.61	15.55–21.68		226.26	177.34–275.17	
	Group effect			Group effect		
BV	−5.62	−8.99 – −2.26	*p* = 0.002** Δ = −130.2%	−74.11	−124.84 – −23.38	*p* = 0.005** Δ = −132.8%
UV	−3.40	−7.14 – 0.34	*p* = 0.073 Δ = −118.3%	−39.86	−93.81 – 14.09	*p* = 0.143 Δ = −117.6%
	Task effect			Task effect		
Sorting	−4.11	−7.05 – −1.16	*p* = 0.009** Δ = −122.1%	−120.75	−166.68 – −74.82	*p* < 0.001*** Δ = −153.4%
Heavy load	3.89	0.26–7.51	*p* = 0.037* Δ = −79.1%	8.53	−40.35 – 57.41	*p* = 0.719 Δ = −96.2%
Stairs	7.13	2.01–12.25	*p* = 0.009** Δ = −61.7%	−8.37	−56.07 – 39.33	*p* = 0.718 Δ = −103.7%
Uneven ground	−1.25	−4.14 – 1.64	*p* = 0.377 Δ = −106.7%	−20.08	−61.01 – 20.85	*p* = 0.317 Δ = −108.9%
Tray	−4.62	−6.85 – −2.39	*p* < 0.001*** Δ = −124.8%	−68.32	−110.30 – −26.34	*p* = 0.003** Δ = −130.2%
Wood beam	−6.62	−9.25 – −3.99	*p* < 0.001*** Δ = −135.6%	−67.62	−115.47 – −19.78	*p* = 0.008** Δ = −129.9%
Inclined plane	−3.18	−4.95 – −1.41	*p* = 0.001** Δ = −117.1%	−34.81	−73.04 – 3.42	*p* = 0.072 Δ = −115.4%
Picture recognition	−1.20	−4.17 – 1.77	*p* = 0.408 Δ = −106.4%	−86.80	−123.40 – −50.21	*p* < 0.001*** Δ = −138.4%
Walk in the dark	−4.43	−6.57 – −2.30	*p* < 0.001*** Δ = −123.8%	−39.76	−70.76 – −8.77	*p* = 0.015* Δ = −117.6%
UTurn	57.14	43.29–71.00	*p* < 0.001*** Δ = 207.0%	46.94	−8.87 – 102.76	*p* = 0.094 Δ = −79.3%
	Group × Task effect			Group × Task effect		
BV × Sorting	3.31	−0.22 – 6.84	*p* = 0.065 Δ = −82.2%	64.14	14.98–113.29	*p* = 0.012* Δ = −71.7%
UV × Sorting	1.40	−2.37 – 5.17	*p* = 0.457 Δ = −92.5%	27.25	−24.04 – 78.54	*p* = 0.289 Δ = −88.0%
BV × Heavy load	−2.60	−6.52 – 1.32	*p* = 0.188 Δ = −114.0%	21.49	−28.53 – 71.50	*p* = 0.390 Δ = −90.5%
UV × Heavy load	−3.18	−7.41 – 1.05	*p* = 0.137 Δ = −117.1%	6.42	−45.34 – 58.17	*p* = 0.803 Δ = −97.2%
BV × Stairs	−0.55	−6.37 – 5.27	*p* = 0.849 Δ = −103.0%	25.80	−25.06 – 76.67	*p* = 0.311 Δ = −88.6%
UV × Stairs	−0.86	−6.68 – 4.97	*p* = 0.767 Δ = −104.6%	5.76	−50.74 – 62.26	*p* = 0.838 Δ = −97.5%
BV × Uneven ground	0.81	−2.36 – 3.97	*p* = 0.609 Δ = −95.6%	26.38	−15.70 – 68.47	*p* = 0.212 Δ = −88.3%
UV × Uneven ground	−0.65	−3.95 – 2.65	*p* = 0.691 Δ = −103.5%	−3.03	−47.92 – 41.86	*p* = 0.892 Δ = −101.3%
BV × Tray	2.19	−0.50 – 4.88	*p* = 0.108 Δ = −88.2%	56.92	14.05–99.79	*p* = 0.011* Δ = −74.8%
UV × Tray	0.78	−2.20 – 3.75	*p* = 0.601 Δ = −95.8%	23.34	−24.94 – 71.61	*p* = 0.334 Δ = −89.7%
BV × Wood beam	3.03	0.06 – 6.00	*p* = 0.046* Δ = −83.7%	40.28	−11.51 – 92.06	*p* = 0.124 Δ = −82.2%
UV × Wood beam	1.57	−1.89 – 5.02	*p* = 0.365 Δ = −91.6%	21.24	−36.06 – 78.55	*p* = 0.458 Δ = −90.6%
BV × Inclined plane	2.89	0.22–5.56	*p* = 0.035* Δ = −84.5%	32.58	−7.24 – 72.40	*p* = 0.106 Δ = −85.6%
UV × Inclined plane	−0.24	−2.82 – 2.34	*p* = 0.850 Δ = −101.3%	2.99	−39.71 – 45.69	*p* = 0.888 Δ = −98.7%
BV × Picture recognition	2.97	−0.83 – 6.77	*p* = 0.122 Δ = −84.0%	63.12	23.15–103.09	*p* = 0.003** Δ = −72.1%
UV × Picture recognition	3.51	−0.24 – 7.25	*p* = 0.066 Δ = −81.1%	37.14	−5.55 – 79.82	*p* = 0.086 Δ = −83.6%
BV × Walk in the dark	1.70	−0.78 – 4.18	*p* = 0.173 Δ = −90.9%	32.60	−2.23 – 67.42	p = 0.066 Δ = −85.6%
UV × Walk in the dark	0.70	−2.11 – 3.51	*p* = 0.617 Δ = −96.2%	3.16	−34.00 – 40.32	*p* = 0.864 Δ = −98.6%
BV × UTurn	−23.84	−41.14 – −6.54	*p* = 0.008** Δ = −228.1%	19.29	−41.43 – 80.02	*p* = 0.524 Δ = −91.5%
UV × UTurn	−10.93	−30.07 – 8.21	*p* = 0.255 Δ = −158.7%	17.03	−59.05 – 93.11	*p* = 0.653 Δ = −92.5%

Results from linear mixed-effects models analyzing angular velocity (deg/s) and angular acceleration (deg/s^2^) of head movements across groups and tasks. The table shows estimates, 95% confidence intervals (CI), *p*-values for main effects and interactions, and percentage difference from the intercept (Δ). Healthy subjects (HS) and Walk task were defined as reference groups. BV: bilateral vestibulopathy patients; UV: unilateral vestibulopathy patients. Group effects represent differences compared to HS, task effects represent differences compared to walk, and interaction effects (Group × Task) represent differential task effects between patient groups and HS. Significance levels: *p < 0.05 (light yellow), **p < 0.01 (yellow), ***p < 0.001 (orange).

For the UV group (Figure 3), results for the different tasks show more dispersion, indicated by a larger inter-quartile range, although angular velocities remain generally between 8 and 12 deg/s, and angular accelerations between 118 and 159 deg/s^2^. Picture recognition, Stairs, Sorting, and Heavy load tasks were located at the extremes of these ranges. As with the BV group, the UTurn task exhibited the highest angular velocities, close to 45 deg/s. Once again, most tasks (except UTurn, Sorting and Wood beam tasks) were distributed within the interquartile range (Q1: 11 deg/s, 115 deg/s^2^; Q3: 31 deg/s, 288 deg/s^2^) of the overall group distribution. Statistical analyses of the group effect (Table 3) showed that the UV group exhibited decreases in angular velocity (estimate = −3.40, 95% CI: [−7.14; 0.34], *p* = 0.073, Δ = −118.3%) and angular acceleration (estimate = −39.86, 95% CI: [−93.81; 14.09], *p* = 0.143, Δ = −117.6%) compared with HS group, but without reaching statistical significance.

Concerning the HS group (Figure 4), the tasks are also distributed between 10 deg/s and 16 deg/s^2^ for angular velocities and 90 to 205 deg/s^2^ for angular accelerations. Some tasks exhibited higher values. For example, Stairs reached angular velocities of around 25 deg/s and angular accelerations of 175 deg/s^2^; Walk and Uneven ground demonstrated angular velocities around 15 deg/s with angular acceleration of 175 deg/s^2^; finally, Heavy load showed angular velocities of around 15 deg/s and angular acceleration of 205 deg/s^2^. As in the other groups, only the UTurn task’s values stand out clearly, with angular velocities of up to 60 deg/s, and angular accelerations of approximately 210 deg/s^2^. Most other tasks and participants remained in the Q1 and Q3 quartiles (Q1: 13 deg/s, 128 deg/s^2^; Q3: 39 deg/s, 358 deg/s^2^) of the overall group distribution.

**Figure 5:**
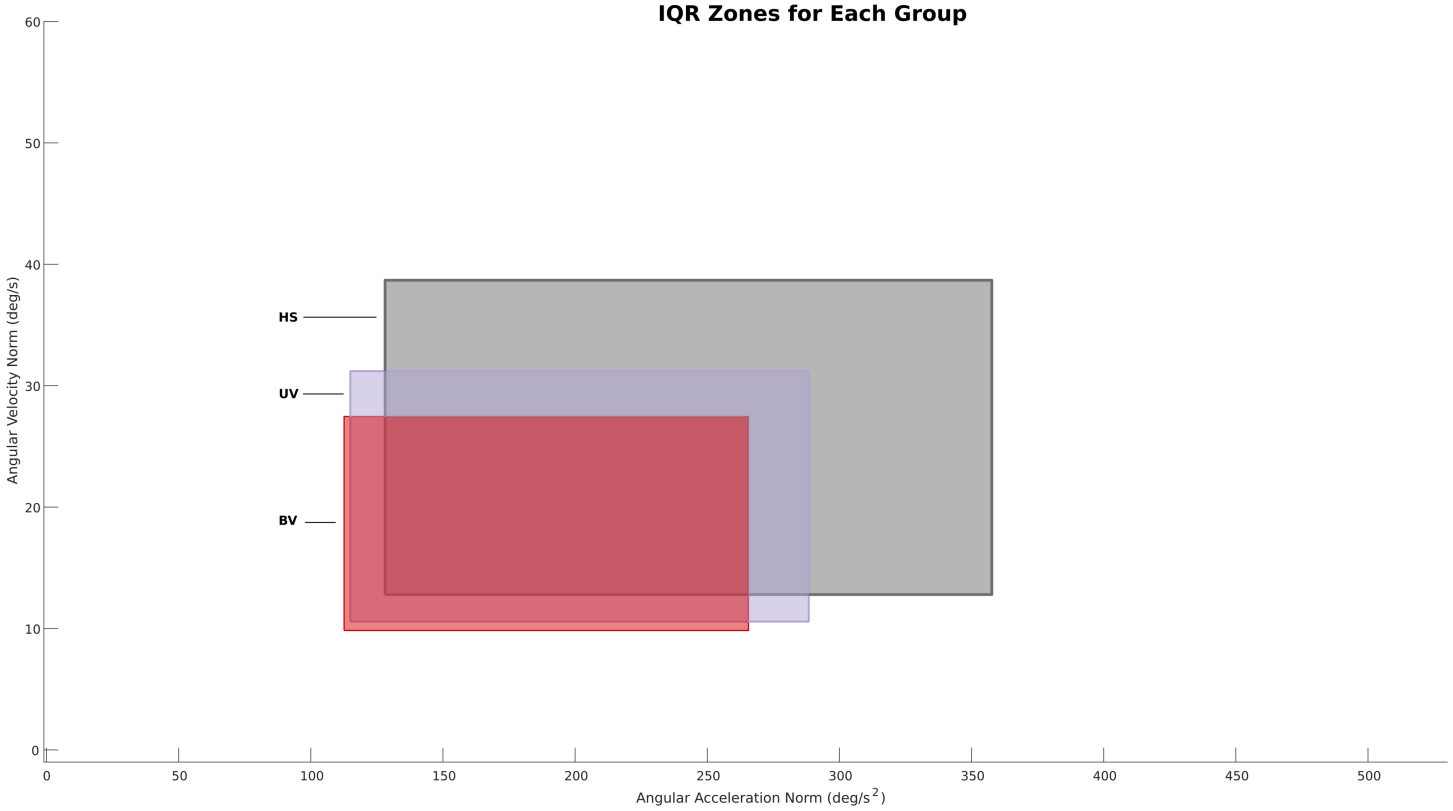
Q1–Q3 surfaces all groups. Red: bilateral vestibulopathy patients, Purple: unilateral vestibulopathy patients, Grey: healthy subjects.

To facilitate comparison of head motion kinematics between groups, Figure 5 presents the Q1-Q3 range for the three participant groups. The Q1 and Q3 values were extracted by group for all tasks analyzed together. A progressive increase in the area between Q1 and Q3 was observed between the pathological groups (BV and UV) and the control group (HS), with respective values for the area of 2.69 deg^2^/s^3^ (BV), 3.58 deg^2^/s^3^ (UV) and 5.97 deg^2^/s^3^ (HS). The UV group also showed slightly higher angular velocities and angular accelerations than the BV group.

**Figure 6:**
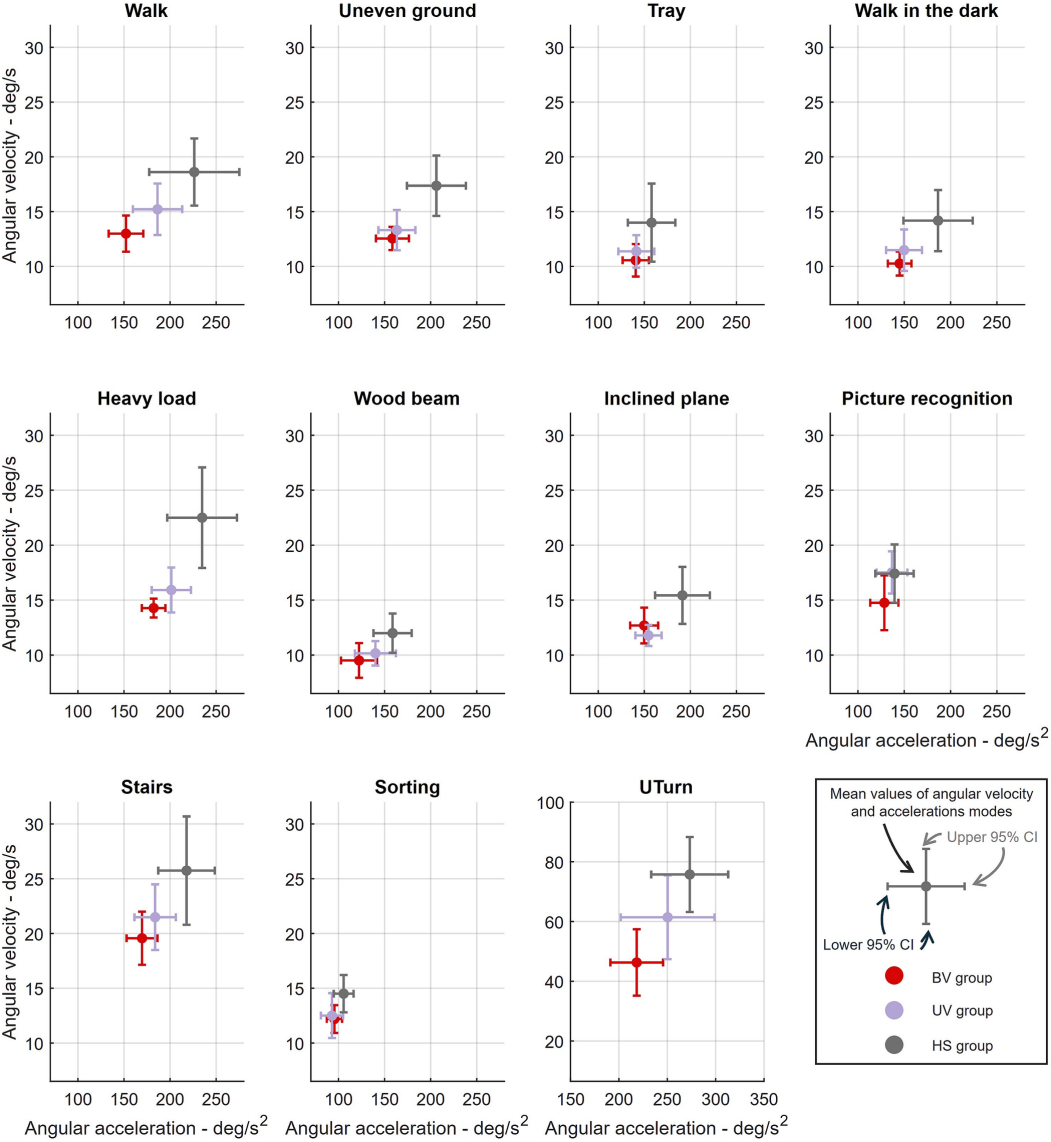
Mean values of angular acceleration (x-axis) and mean angular velocity (y-axis) modes for each group and each task. Each panel represents one task. Circles indicate the group mean; error bars show the 95% confidence interval. Data were calculated for each group within each task. BV: Bilateral vestibulopathy patients (red), UV: unilateral vestibulopathy patients (purples), HS: healthy subjects (grey), CI: confidence interval.

Finally, the task-by-task comparison, illustrated in Figure 6, and the statistical results, detailed in Table 3, showed that certain tasks consistently modulated head movement dynamics across all groups. The Sorting task showed a reduction in both angular velocity and acceleration, while the Tray, Wood beam, Inclined plane, Walk in the dark, and Picture recognition tasks showed reduced angular acceleration. Conversely, the UTurn task greatly increased angular velocity without significantly affecting angular acceleration, and the Stairs task increased angular velocity with stable angular acceleration. Some group × task interactions were significant: the BV group showed slight increases in angular velocity for the Wood beam and Inclined plane tasks, while for the UTurn task, a significant reduction in angular velocity was observed. For angular acceleration, the BV group showed significant positive effects for the Sorting, Tray, and Picture recognition tasks. Other interactions were either non-significant or showed moderate variations without exceeding the significance threshold.

**Figure 7:**
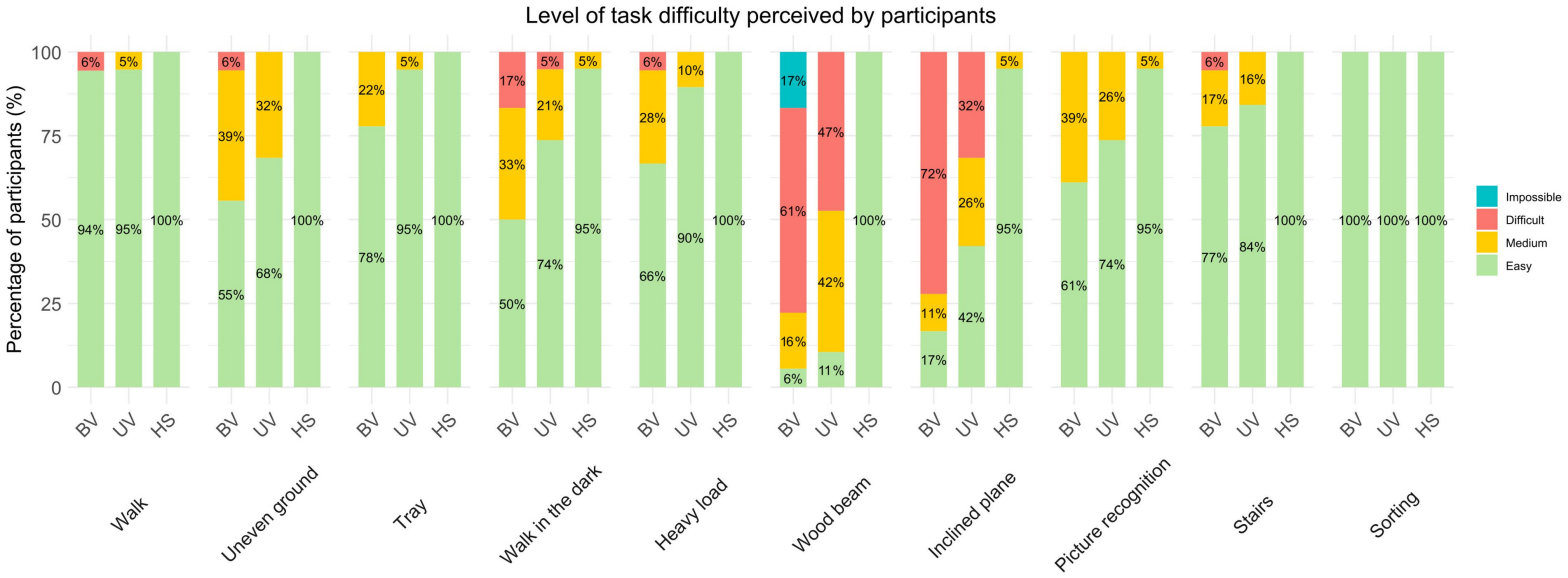
Level of task difficulty perceived by participants, percentage by group. BV: bilateral vestibulopathy patients, UV: unilateral vestibulopathy patients, HS: healthy subjects.

To complete this study, patients’ subjective perception of task difficulty was assessed (Figure 7). 61% of BV patients considered the Wood beam task difficult, and 17% were unable to perform it. Among UV patients, 47% also rated this task as difficult, while all HS subjects found it easy. The second most frequently perceived difficult task was the Inclined plane, with 72% of BV and 32% of UV patients describing it as difficult. Finally, the Walk in the dark task performed was considered difficult by 17% of BV patients. To complement these results, weak and non-significant correlations were observed between subjective values of perceived difficulty and values of angular velocity and angular acceleration modes (Supplementary material 1).

## 4 Discussion

The aim of this study was to evaluate head movements while performing common functional mobility tasks in a semi-standardized environment in patients with BV and UV, and to compare them with the HS group. We further aimed at characterizing the head movement strategies adopted by each of these groups. The results obtained demonstrate significant differences in head movement patterns between groups, with the HS group showing higher overall head angular velocities and angular accelerations than the pathological groups, suggesting greater freedom of head movements.

### 4.1 Head movement strategies

We observed greater variations in angular velocities and smaller variations in angular accelerations between the groups, which can be partly explained by the time taken (Supplementary material 2) to perform the tasks (Picture recognition, Stairs, Sorting, and UTurn tasks). In other words, since angular accelerations recorded during the active rotation phase were similar between groups, this suggests a preserved capacity to generate motor effort of the same intensity, but in the context of an overall more cautious strategy as reflected by the larger variations in angular velocities.

Overall, BV patients showed a drastic reduction in head angular velocity and angular acceleration during walking and across most functional mobility tasks, both as a main group effect and for specific tasks such as Tray, Wood beam, and Walk in the dark, confirming a global restriction strategy. Interactions between group and task highlighted that for certain contexts (e.g., BV × Wood beam, BV × Tray), the reduction was less pronounced, suggesting some adaptations from the patients. The BV patients appeared to adopt a more rigid posture, between the head and the trunk during complex tasks, as observed in previous studies (Pozzo et al., 1991; Grouvel et al., 2024), thus relying more on visual and proprioceptive information.

Similar trends were observed in UV patients, although behavior closer to that of HS was expected. In a previous study, we demonstrated that around 70% of UV patients achieve performance similar to that of HS (Boutabla et al., 2025). In the current results, as hypothesized, only a trend for reduced head movements in UV was observed, without significance for most tasks, except for Heavy load and Picture recognition (angular velocity), possibly due to the heterogeneity of this group (Karabulut et al., 2023) and the diversity of included tasks. All of this could suggest that one functioning vestibular system seems to provide enough information to allow for relative preservation of motor strategies and/or promote compensatory strategies. However, the most demanding tasks, such as UTurn, tend to accentuate the dissociation between the groups.

Finally, most BV and UV patients performed tasks with angular velocities around 10 deg/s and accelerations between 85 and 150 deg/s^2^. These relatively low values compared with the HS group support the hypothesis of cautious motor behavior (Huppert et al., 2020), and the adoption of different compensatory head movements (Pozzo et al., 1991; Mamoto et al., 2002; Grouvel et al., 2024). This characteristic may also be associated with a greater head-torso variability and altered head movements in patients, as shown by Zobeiri et al. (2021) and Zobeiri et al. (2022). Nevertheless, BV and UV patients showed task-specific head movement patterns indicating their ability to adjust their head movements to accomplish the task. The reduced and more stable head movements observed in BV and UV patients in our study reflect this same principle, probably a protective and compensatory mechanism (Pozzo et al., 1991; Pozzo et al., 1990). These results are reinforced by complementary analyses, which reproduce those of the main study but obtained from the trunk sensor (Supplementary material 3). In this complementary analysis, the different tasks are clearly distinguished within each group. Furthermore, analysis of the interquartile areas (Q1–Q3) reveals a strong similarity between the BV and UV groups, whose areas overlap almost entirely and remain very close to those observed in HS. These observations suggest that, regardless of vestibular status, trunk movements remain comparable between groups, while head stabilization strategies differ depending on the type of vestibular disorder.

The HS group showed well-differentiated head movement patterns depending on the task, indicating their ability to adjust their head movements more precisely (Pozzo et al., 1995). However, it is noteworthy that these participants showed greater behavioral changes during more complex tasks. For example, during walking with a tray, they also reduced their head angular velocities, adopting a pattern similar to the one observed in both BV and UV patient groups. The differences between head movement patterns would be reduced during the most complex tasks. This demonstrated a natural adaptive response of the human body in situations perceived as complex or insecure (Young and Mark Williams, 2015).

Among all the tasks analyzed, the UTurn is characterized by particularly high angular velocities, especially in HS. These maximum values represent key information for defining realistic reference thresholds, considerations that are particularly important for the design of innovative devices such as the vestibular implant. This study showed that most of the predominant values per task occur at relatively low velocities and accelerations which might help determine useful input ranges for the sensors associated with these devices.

This study provides an overview of participants’ head movements patterns, highlighting particularly the differences between BV patients and the HS group. These findings could also provide concrete information to guide rehabilitation strategies and technical developments, whether to restore vestibular function with a vestibular implant (Guinand et al., 2015; Vesper et al., 2024) or to reduce symptom severity with vestibular physiotherapy. This will make it possible to target the limitations observed in functional mobility activities in order to optimize exercises, using reduced ranges of motion and taking into account the patient’s actual abilities and apprehensions. These results could guide the adjustment of vestibular stimulation parameters to reproduce as closely as possible the head movements observed in HS (e.g., by selecting the correct acceleration amplitude for the stimulation parameters used to fit vestibular implants), as well as the choice of exercises designed to train patients’ head movements.

### 4.2 Objective and subjective correlations

It is interesting to note that the objective results concerning head movements do not always correspond to the subjective assessments of task difficulty, whether in patients or in HS, which is confirmed by the analysis of correlations between subjective and objective values. For the tasks considered most difficult by patients – the Wood beam, Walk in the dark, and Inclined plane tasks—there was a slight decrease in angular velocities and angular accelerations in all three groups, whereas healthy subjects did not consider these tasks to be particularly difficult. Conversely, certain tasks such as walking, walking on an uneven ground, walking while looking and recognizing pictures, climbing up and down stairs, and walking and making a U-turn were not perceived as difficult by patients, although they revealed marked differences in head movements compared with healthy subjects. The differences obtained for comfortable walking are still hard to explain, but it can be hypothesized that this is due to a reduction in walking speed, i.e., patients take longer to complete the task (Supplementary material 2), in patients compared with healthy subjects (Boutabla et al., 2025; Grouvel et al., 2025b). Overall, the BV group gave the lowest subjective rating percentage of “easy,” reflecting the difficulty perceived by the patients in following this protocol, although no correlation with objective head movement values was found. Patients may find a task difficult when they are unable to adapt their motor profile correctly to the task in question. Tasks for which they are able to implement an appropriate motor strategy (different depending on whether they have a pathology) are not considered difficult because they are able to perform them even if the motor response is not considered “ideal” (compared to healthy subjects).

The poor correlations observed between angular velocities and angular accelerations suggest no linear dependence, reflecting the complex nature of human movement patterns, particularly when compensatory strategies are present. This finding justified our approach of analyzing both parameters independently and supports the hypothesis that vestibular dysfunction affects different aspects of head movement control through distinct mechanisms. A Fourier transform frequency analysis could also have been relevant to provide information based on the types of movements performed, as head rotations have a frequency range between 0.5 and 5.0 Hz (Grossman and Leigh, 1990). However, the non-periodic, non-predictable, and heterogeneous nature of the head movements (Grossman and Leigh, 1990) recorded made this approach difficult to implement for this study.

### 4.3 Methodological considerations and perspectives

By focusing on functional mobility activities, this study offered a more ecologically valid representation of patients’ everyday experiences, and enabled a more general analysis than when conducted in the laboratory (Mijovic et al., 2014; Wuehr et al., 2022). However, it also introduced variability due to less standardization in task performance.

Furthermore, the use of linear mixed models enabled a precise estimation of group, task, and interaction effects on angular velocity and angular acceleration parameters and allowed to quantify the magnitude of group differences beyond mean comparisons, with adjusted confidence intervals and *p*-values for each effect. Our methodology differed from previous work (Mijovic et al., 2014) by considering all three components of the inertial sensors to eliminate dependence on movement plane and capture global head movement. This approach provided a more complete representation of head movements in space, though it precluded analysis of direction-specific movements such as pitch or yaw plane rotations, which could have provided additional insights into specific compensatory strategies.

Finally, the slight difference observed in the range of head movements between BV and UV patients suggests that even small improvements in head stabilization strategies could already have a positive clinical impact on the quality of life of BV patients. In fact, patients with UV generally have a better quality of life than those with BV (Guinand et al., 2012; Karabulut et al., 2024; Sun et al., 2014). However, the minimum change required for a patient to perceive an improvement in their quality of life remains to be determined.

### 4.4 Limitations

This study classified UV and BV participants based on lateral-canal VOR function, consistent with the Bárány Society criteria, while healthy subjects were required to demonstrate normal function across all six canals. Although six-canal vHIT data were collected in all participants, vertical-canal and otolith measures were not used diagnostically, leaving the possibility of undetected vestibular heterogeneity across groups. Furthermore, vHIT was used instead of calorics in UV patients due to superior tolerability, but this may limit sensitivity to low-frequency deficits. Finally, the absence of consensus criteria for chronic unilateral vestibulopathy places inherent limitations on the generalizability of our UV classification approach.

Factors that could influence motor performance and head movement, such as regular physical activity, age, or access to vestibular rehabilitation (Hall et al., 2016), were not controlled when participants were recruited, and were not considered during the analysis. However, we provided participants’ information (Supplementary material 4) on their disease and rehabilitation in order to provide a comprehensive overview of the cohort and better understand patient compensation. This heterogeneity could explain some of the intra- and inter-group variability. In addition, the UTurn task was extracted from a task comprising other movements (walking with a Heavy load) and other instructions, which may have led to different execution strategies between individuals, thus limiting comparability. The results should therefore be interpreted with caution. Finally, the data were acquired over a wide time range, in a semi-standardized environment open to the outside, and the weather in different seasons may have had an impact on patients’ performance in carrying out the protocol, as well as on the interactions with the operators during the completion of tasks. However, the groups were recruited in a broadly equivalent and random manner throughout the experimental period.

## 5 Conclusion

This study analyzed head movements during functional mobility tasks, revealing the functional correlate of vestibulopathy. Patients with bilateral vestibulopathy, and to a lesser extent patients with unilateral vestibulopathy exhibit reduced angular velocities of the head, with strong and significant effects observed for both group and task main effects, as well as specific group × task interactions in the linear mixed model analysis. This reduction was most pronounced in BV patients and for complex tasks (e.g., Tray, Wood beam, Walk in the dark). In contrast, UV patients showed only moderate, mostly non-significant, changes compared to healthy subjects. These alterations probably reflected compensatory mechanisms aimed at minimizing the impact of the vestibular deficit, but also the fact that one ear could provide enough information to perform motor tasks and/or develop effective compensation strategies. This would be entirely consistent with a unilateral vestibular implant, where most symptoms could be reduced. Moreover, angular accelerations remained relatively preserved and generally not significantly different between controls and patients except for isolated tasks, suggesting that while rapid movements can be generated, overall movement amplitude is restricted through a cautious strategy. Indeed, tasks such as UTurn, stair climbing, walking on unstable ground, or simple walking resulted in significant differences between groups.

These findings could provide guidance for therapeutic interventions, such as vestibular physiotherapy, enabling clinicians to design targeted exercises that progressively challenge patients’ restricted movement ranges. For vestibular implant development, these data could provide realistic stimulation targets based on actual functional movement. Furthermore, these movement profiles offer an objective framework for monitoring patients’ functional status over time, allowing clinicians to track rehabilitation progress and adjust treatment strategies accordingly.

Although the semi-standardized environment closely approximates real-life conditions, future studies should extend this approach to fully naturalistic environments using wearable sensors during patients’ actual real-life activities. This would provide even more ecologically valid information about the impact of vestibular dysfunction. Furthermore, coupling these movement profiles with data from this study could enable the creation of comprehensive ‘vestibulograms’ that would establish a direct correlation between vestibulopathy and specific task-related limitations. Such information would improve our understanding of these deficits.

## Data Availability

Data are presented in a Data Descriptor article (Grouvel et al., 2025a). All data files are also available online on a Zenodo 565 database: https://doi.org/10.5281/zenodo.15081742.
