# Supplementary material for: Characterization of head movement patterns in patients with bilateral and unilateral vestibulopathy during functional mobility tasks
Source: Front Neurosci. 2026 Feb 11;20:1731221. doi: 10.3389/fnins.2026.1731221 (PMC12932612; doi:10.3389/fnins.2026.1731221)
Supplement: Supplementary file 1 [file Data_Sheet_1.docx]

Supplementary Material 1

#
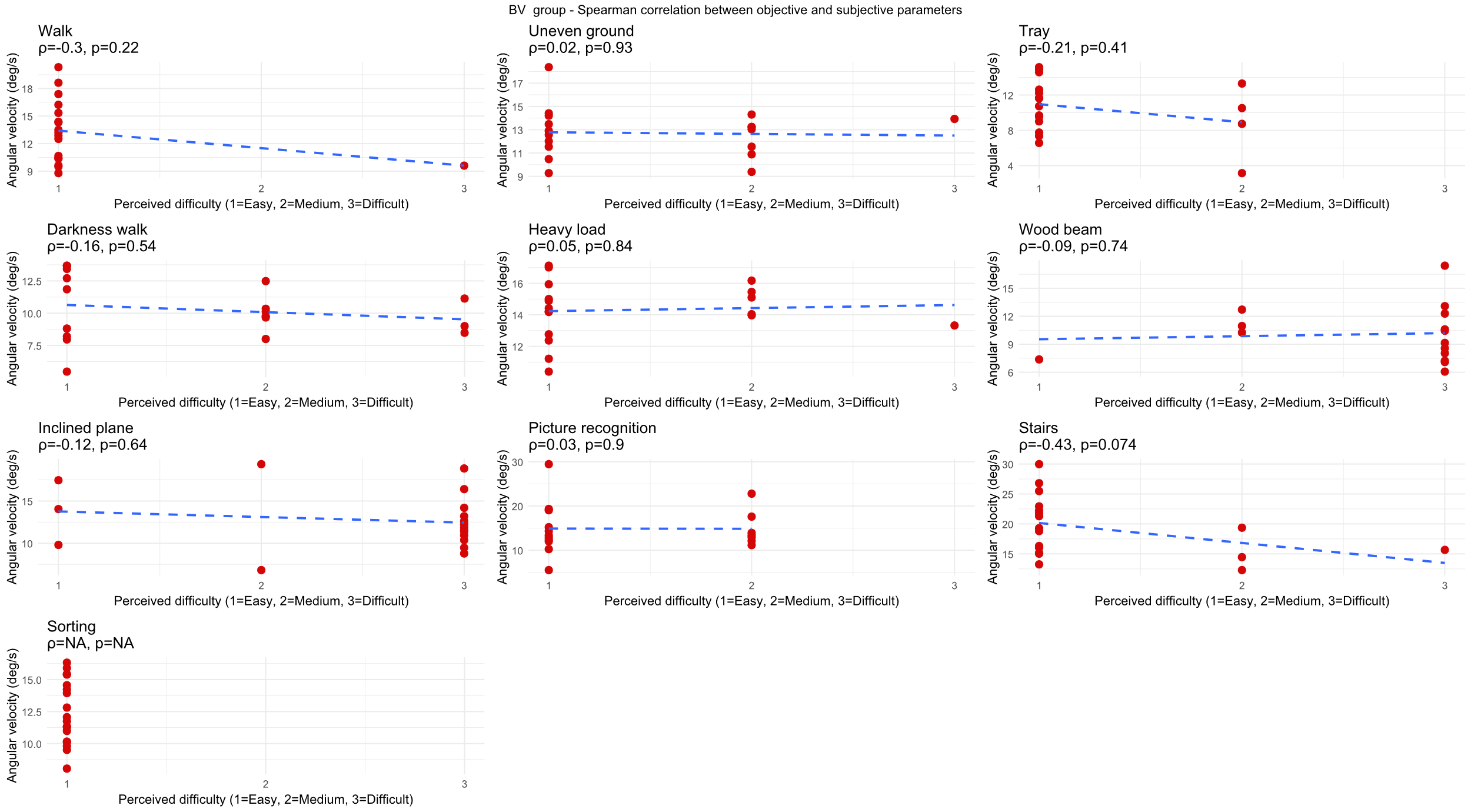
Supplementary Figures

Supplementary Figure 1. Spearman correlation between angular velocity (deg/s) mode values and task difficulty perceived by bilateral vestibulopathy (BV) patients.


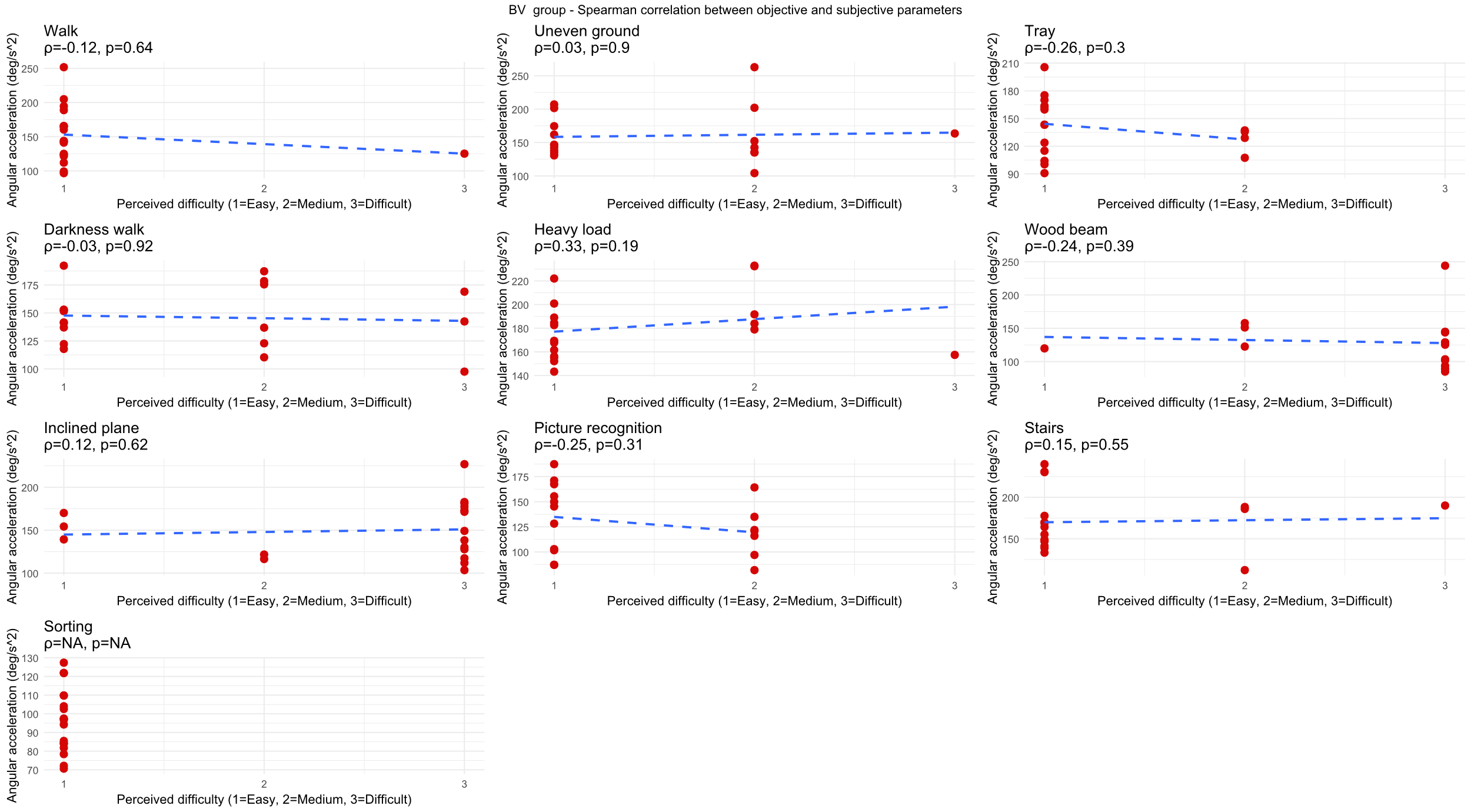


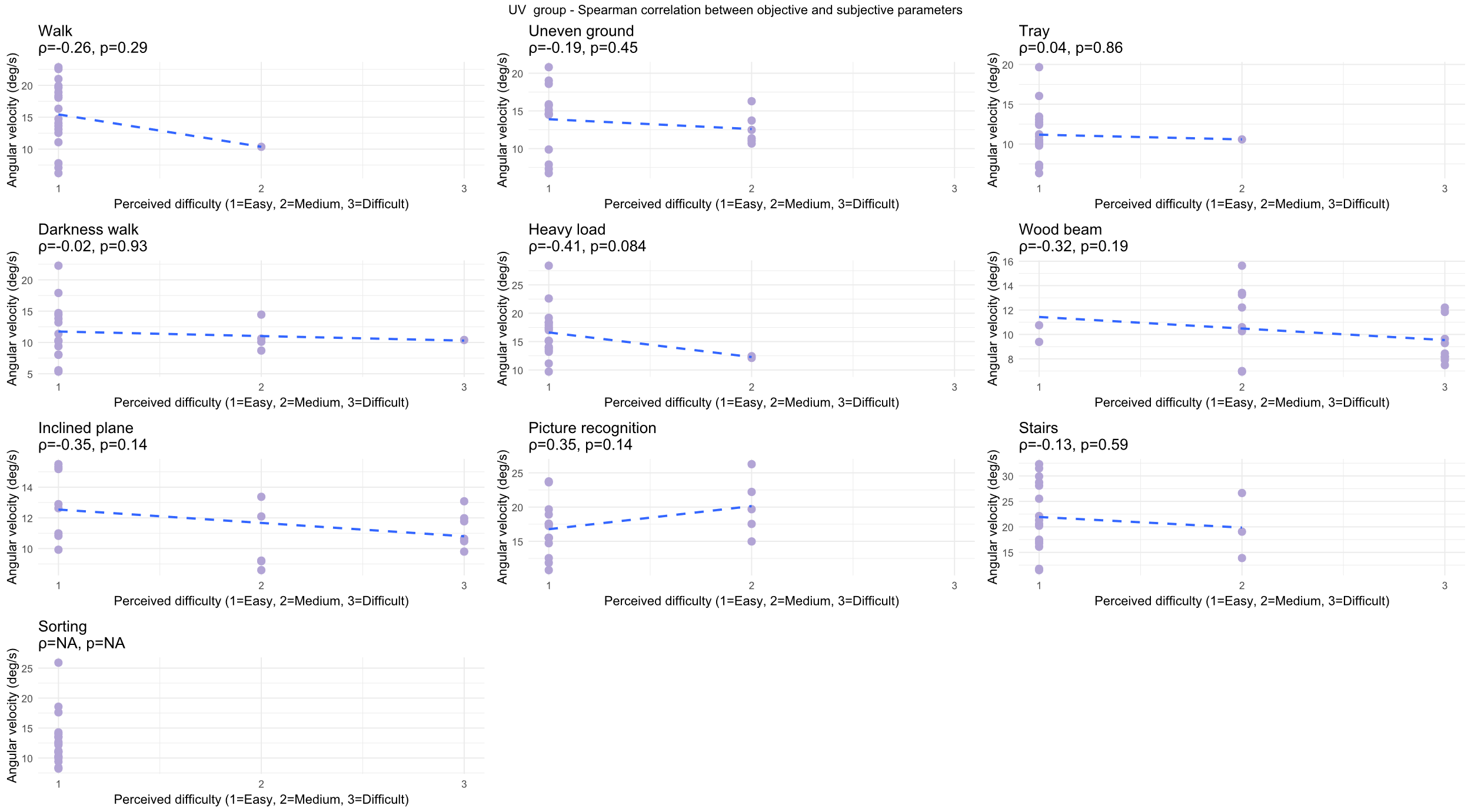


Supplementary Figure 2. Spearman correlation between angular acceleration (deg/s2) mode values and task difficulty perceived by bilateral vestibulopathy (BV) patients.

Supplementary Figure 3. Spearman correlation between angular velocity (deg/s) mode values and task difficulty perceived by unilateral vestibulopathy (UV) patients.


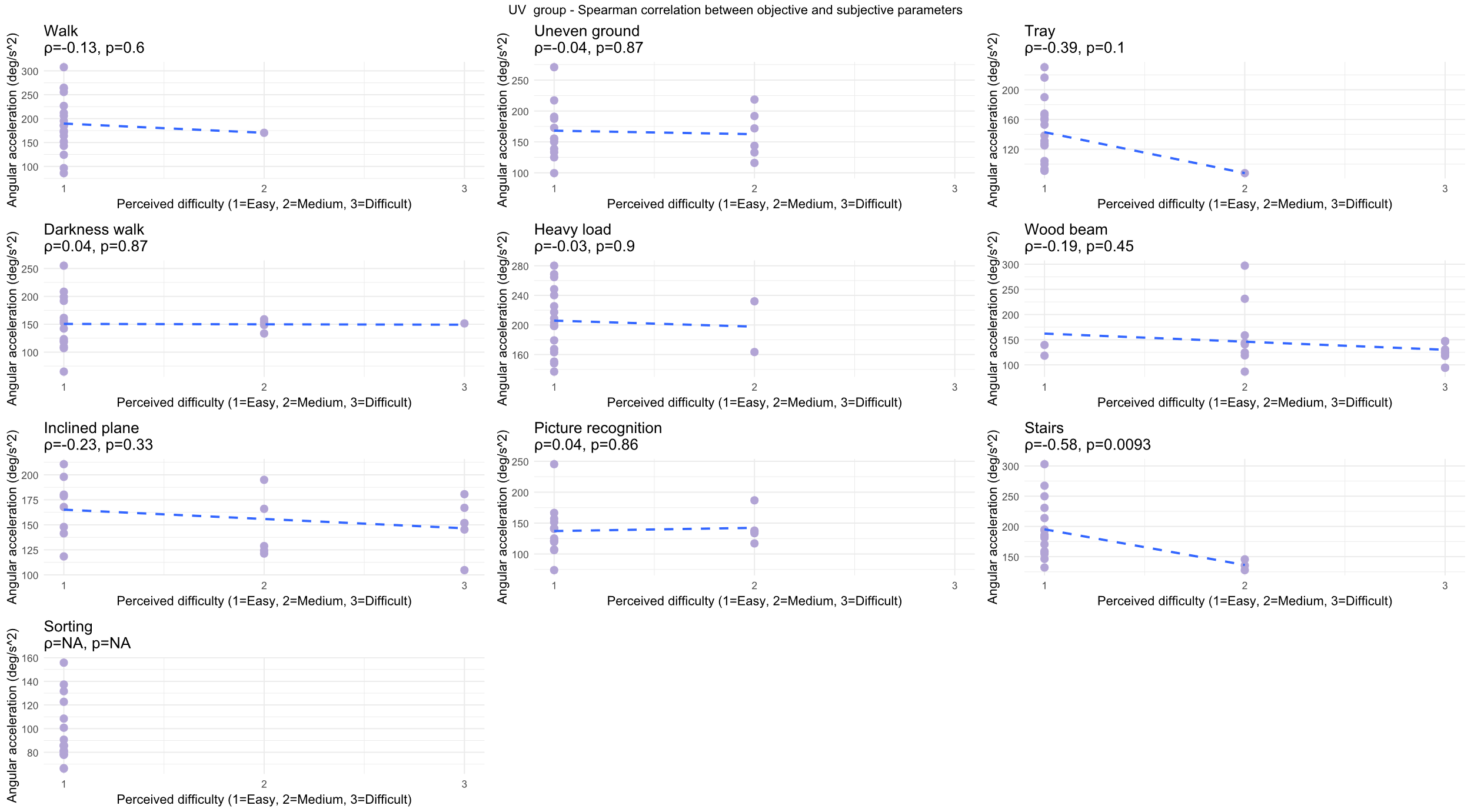


Supplementary Figure 4. Spearman correlation between angular acceleration (deg/s2) mode values and task difficulty perceived by unilateral vestibulopathy (UV) patients.
