# Supplementary material for: Characterization of head movement patterns in patients with bilateral and unilateral vestibulopathy during functional mobility tasks
Source: Front Neurosci. 2026 Feb 11;20:1731221. doi: 10.3389/fnins.2026.1731221 (PMC12932612; doi:10.3389/fnins.2026.1731221)
Supplement: Supplementary file 2 [file Data_Sheet_2.docx]

Supplementary Material 2

# Supplementary Table

| **Task** | **Parameter** | **Kruskal-Wallis (p-value)** | **Post-Hoc Dunn – Holm correction (p-value)** | | | |
| --- | --- | --- | --- | --- | --- | --- |
|  |  |  | **BV - HS** | **UV - HS** | **BV - UV** |  |
| Sorting | *Task time* | 0.124 | - | - | - |  |
|  | *Perceived difficulty* | 1.000 | - | - | - |  |
| Heavy load | *Task time* | 0.028* | 0.022* | 0.188 | 0.345 |  |
|  | *Perceived difficulty* | 0.011* | 0.010** | 0.358 | 0.089 |  |
| Stairs | *Task time* | 0.057 | - | - | - |  |
|  | *Perceived difficulty* | 0.098 | - | - | - |  |
| Uneven ground | *Task time* | 0.001** | < 0.001*** | 0.025* | 0.264 |  |
|  | *Perceived difficulty* | 0.005** | 0.004** | 0.052 | 0.328 |  |
| Tray | *Task time* | 0.331 | - | - | - |  |
|  | *Perceived difficulty* | 0.046* | 0.050* | 0.565 | 0.142 |  |
| Walk | *Task time* | 0.631 | - | - | - |  |
|  | *Perceived difficulty* | 0.575 | - | - | - |  |
| Wood beam | *Task time* | < 0.001*** | < 0.001*** | 0.014* | 0.008** |  |
|  | *Perceived difficulty* | < 0.001*** | < 0.001*** | < 0.001*** | 0.196 |  |
| Inclined plane | *Task time* | < 0.001*** | < 0.001*** | 0.001** | 0.081 |  |
|  | *Perceived difficulty* | < 0.001*** | < 0.001*** | 0.006** | 0.036* |  |
| Picture recognition | *Task time* | 0.013* | 0.179 | 0.304 | 0.019* |  |
|  | *Perceived difficulty* | 0.044* | 0.041* | 0.232 | 0.367 |  |
| Walk in the dark | *Task time* | 0.089 | - | - | - |  |
|  | *Perceived difficulty* | 0.006** | 0.005** | 0.140 | 0.179 |  |

Table 1. Results of the Kruskal-Wallis statistical test for task time and perceived difficulty parameters, followed by the results of Dunn's post-hoc test with Holm correction. * p < 0.05; ** p < 0.01; *** p < 0.001.


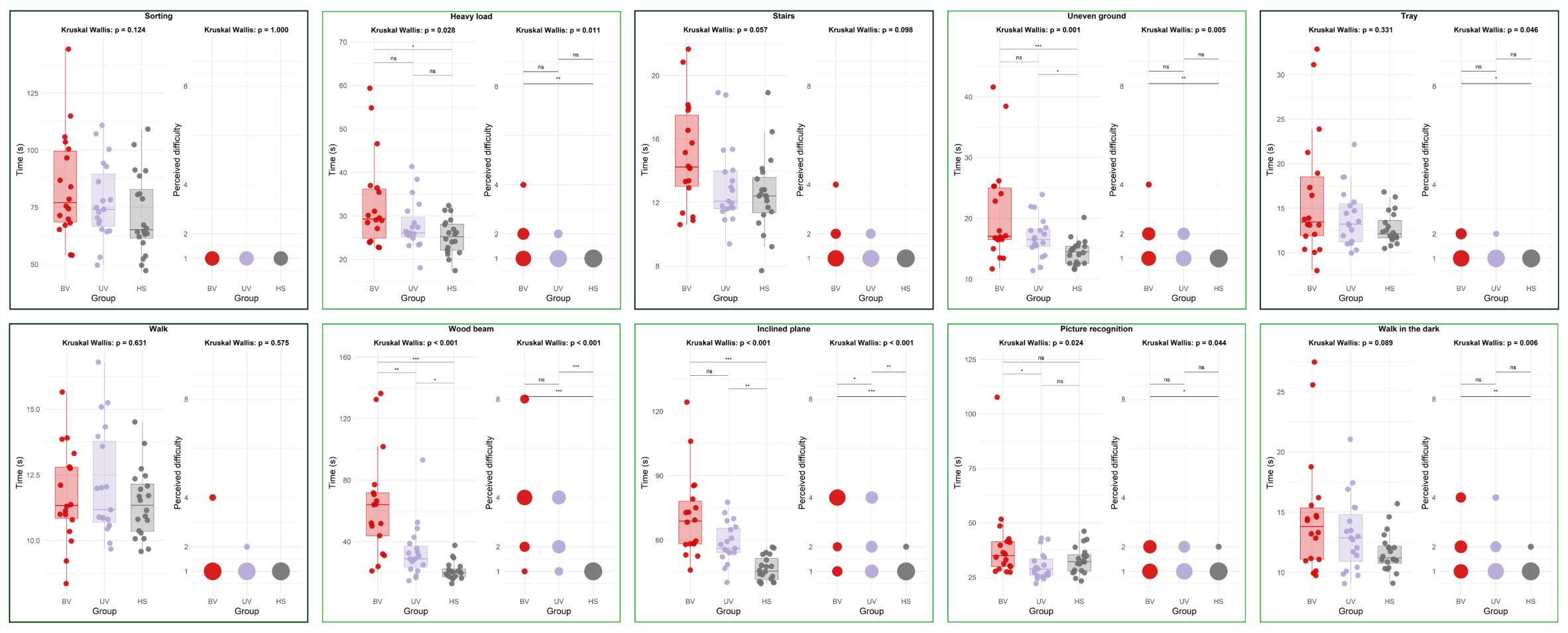


Figure 1. Box plots of task time and perceived difficulty parameters. * p < 0.05; ** p < 0.01; *** p < 0.001. BV: Bilateral vestibulopathy patients; UV: Unilateral vestibulopathy patients; HS: Healthy subjects
