# Supplementary material for: Characterization of head movement patterns in patients with bilateral and unilateral vestibulopathy during functional mobility tasks
Source: Front Neurosci. 2026 Feb 11;20:1731221. doi: 10.3389/fnins.2026.1731221 (PMC12932612; doi:10.3389/fnins.2026.1731221)
Supplement: Supplementary file 3 [file Data_Sheet_3.docx]

Supplementary Material 3

Supplementary Figure 1. Angular acceleration-velocity diagram for daily living tasks for bilateral vestibulopathy patients calculated with the trunk sensor, "Vestibulogram". *Left and bottom: Histogram of distribution for one group and all tasks. Middle: Angular acceleration-velocity diagram with wider colored symbols (one task, all participants of the group) and the small symbols (one task, one participant).*

**
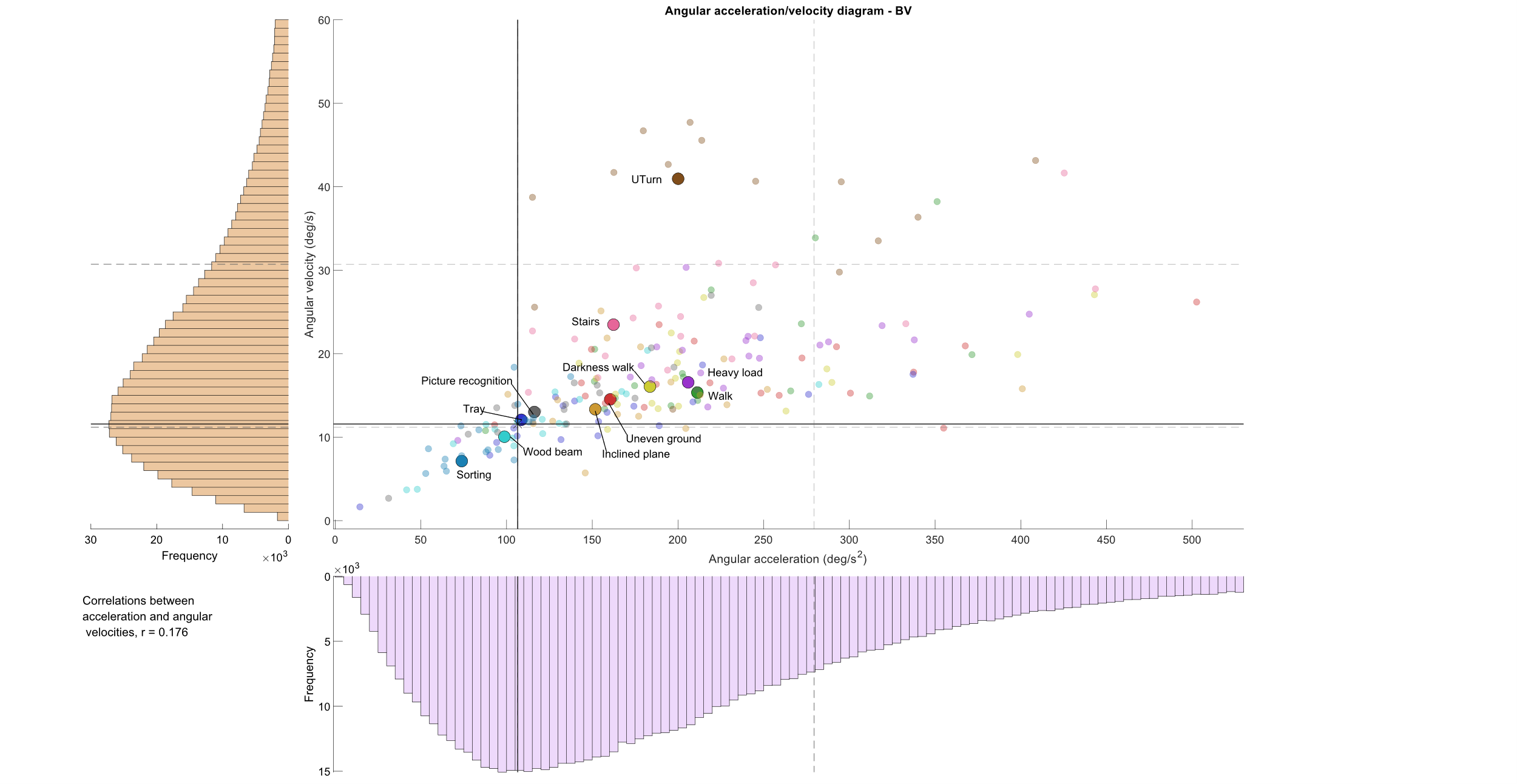
**

Supplementary Figure 2. Angular acceleration-velocity diagram for daily living tasks for unilateral vestibulopathy patients calculated with the trunk sensor, "Vestibulogram". *Left and bottom: Histogram of distribution for one group and all tasks. Middle: Angular acceleration-velocity diagram with wider colored symbols (one task, all participants of the group) and the small symbols (one task, one participant).*

**
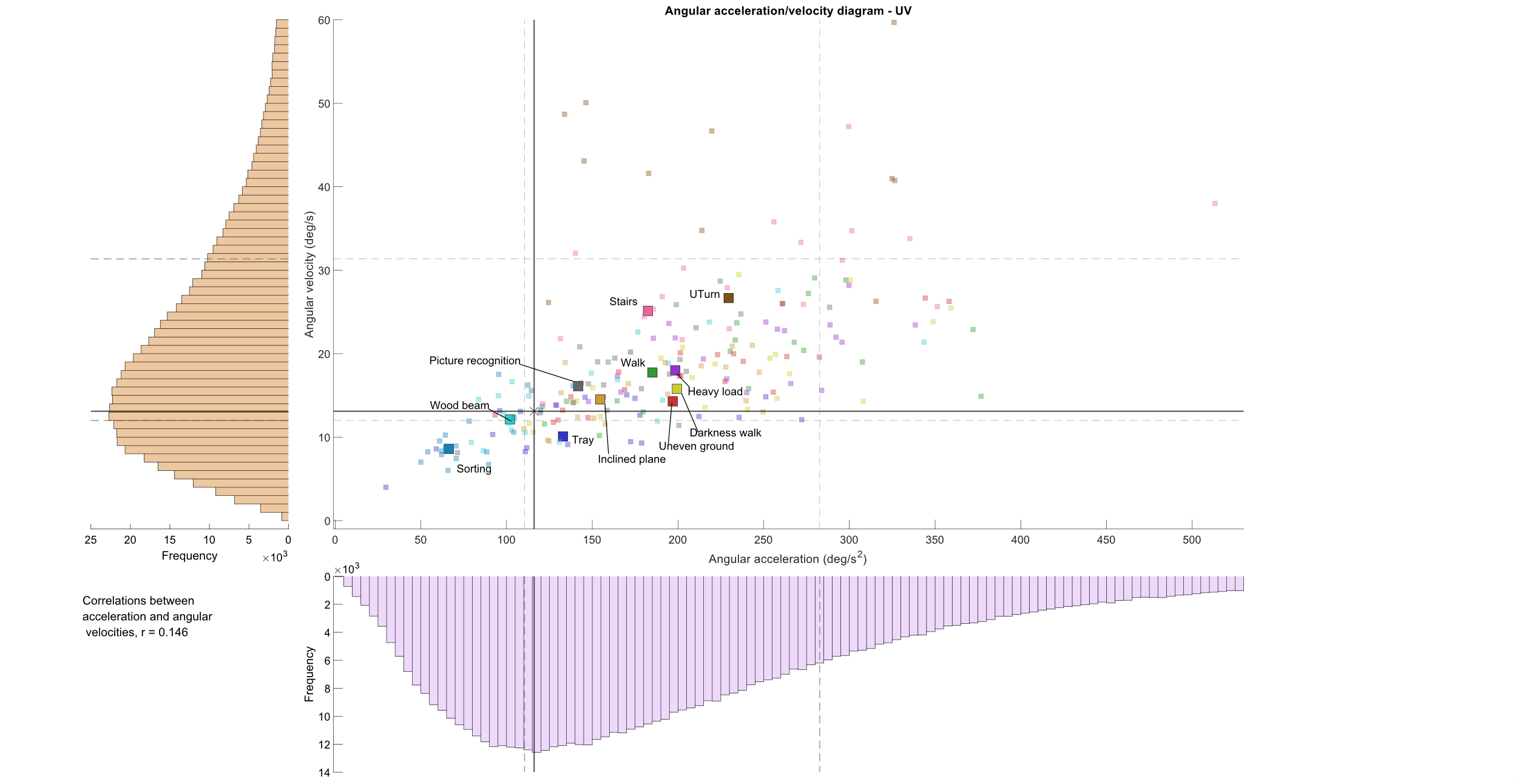
**

**
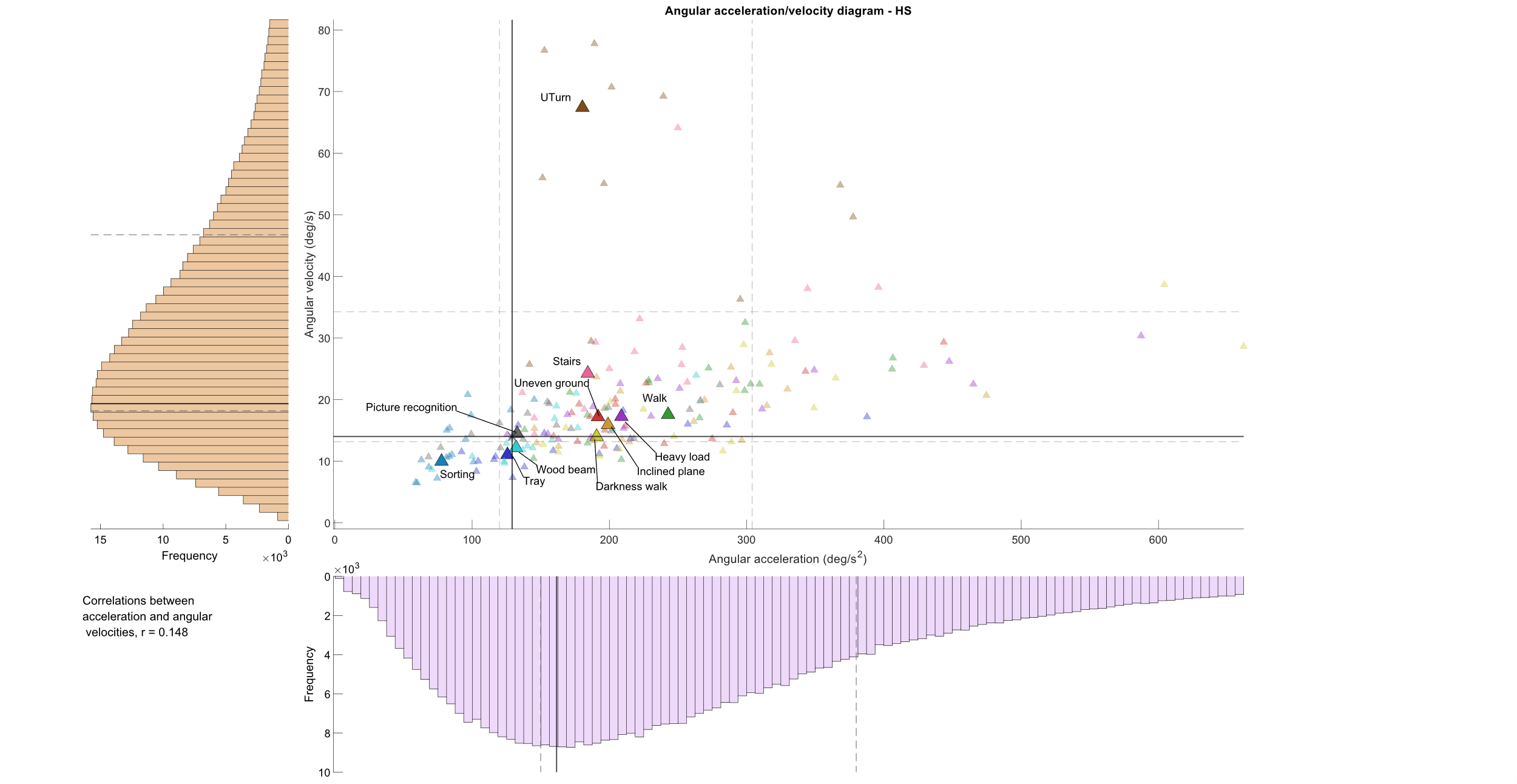
**

Supplementary Figure 3. Angular acceleration-velocity diagram for daily living tasks for healthy subjects calculated with the trunk sensor, "Vestibulogram". *Left and bottom: Histogram of distribution for one group and all tasks. Middle: Angular acceleration-velocity diagram with wider colored symbols (one task, all participants of the group) and the small symbols (one task, one participant).*

**
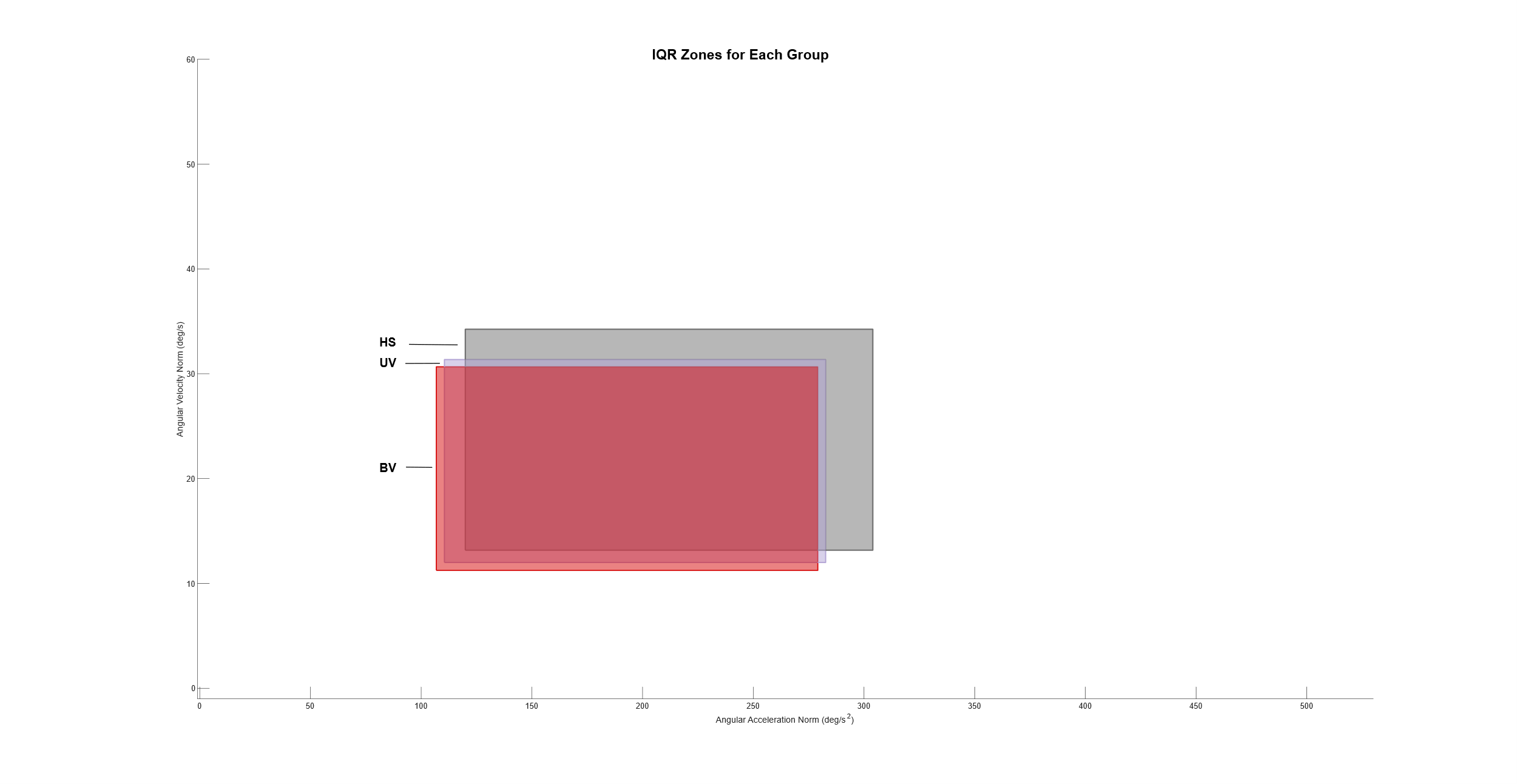
**

Supplementary Figure 4. Q1-Q3 surfaces all groups calculated with the trunk sensor. *Red: Bilateral vestibulopathy patients, Purple: Unilateral vestibulopathy patients, Grey: Healthy subjects.*
