# Supplementary material for: Characterization of head movement patterns in patients with bilateral and unilateral vestibulopathy during functional mobility tasks
Source: Front Neurosci. 2026 Feb 11;20:1731221. doi: 10.3389/fnins.2026.1731221 (PMC12932612; doi:10.3389/fnins.2026.1731221)
Supplement: Supplementary file 4 [file Data_Sheet_4.docx]

Supplementary Material 4

| **Participant** | **Group** | **Sex** | **Etiology** | **Affected side** | **DHI score** | **Date of symptom onset** | **Diagnostic delay (months)** | **Vestibular physiotherapy** | **Physical activity BEFORE disease** | **Physical activity AFTER disease** |
| --- | --- | --- | --- | --- | --- | --- | --- | --- | --- | --- |
| Participant_01 | BV | F | Idiopathic | Both | 66 | 2024 | ~1 | yes | no | NA |
| Participant_02 | BV | M | Schanomma | Both | 56 | 2021 | 30 | no | yes | yes |
| Participant_03 | BV | M | Idiopathic | Both | 64 | 2009 | 5 | yes | yes | yes |
| Participant_04 | BV | M | Idiopathic | Both | 48 | 2013 | 96 | yes | yes | yes |
| Participant_05 | BV | F | Idiopathic | Both | 46 | 2015 | 24 | yes | yes | yes |
| Participant_06 | BV | F | Ototoxic | Both | 74 | 2011 | 0.75 | yes | no | NA |
| Participant_07 | BV | M | Idiopathic | Both | 60 | 2018 | 6 | no | yes | yes |
| Participant_08 | BV | F | Idiopathic | Both | 68 | 2019 | 3 | yes | yes | no |
| Participant_09 | BV | M | Ototoxic | Both | 44 | NA | 3 | no | no | NA |
| Participant_10 | BV | F | Ototoxic | Both | 48 | 1983 | NA | yes | yes | yes |
| Participant_11 | BV | F | Meniere | Both | 70 | 2008 | 156 | yes | yes | yes |
| Participant_12 | BV | F | Idiopathic | Both | 22 | 2022 | 0 | yes | yes | no |
| Participant_13 | BV | M | Cogan syndrome | Both | 26 | 2012 | 1 | no | yes | yes |
| Participant_14 | BV | M | Idiopathic | Both | 12 | 2010 | 2 | yes | yes | yes |
| Participant_15 | BV | M | Idiopathic | Both | 52 | 2010 | 42 | yes | yes | NA |
| Participant_16 | BV | F | Idiopathic | Both | 64 | 2014 | 60 | yes | no | yes |
| Participant_17 | BV | F | Idiopathic | Both | 48 | 2009 | 60 | yes | yes | yes |
| Participant_18 | BV | F | Idiopathic | Both | 22 | NA | NA | yes | yes | no |
| Participant_19 | BV | F | Idiopathic | Both | 20 | 1989 | 276 | yes | yes | yes |
| Participant_20 | UV | F | Meniere | L | 44 | 2005 | 96 | yes | yes | yes |
| Participant_21 | UV | M | Schanomma | L | 20 | 2021 | NA | yes | yes | no |
| Participant_22 | UV | M | Schanomma | R | 44 | 2014 | 2 | yes | yes | no |
| Participant_23 | UV | M | Idiopathic | L | 6 | 2019 | 0 | yes | yes | no |
| Participant_24 | UV | F | Idiopathic | R | 16 | 2019 | 0 | yes | yes | no |
| Participant_25 | UV | F | Schanomma | L | 42 | 2018 | 3 | no | no | NA |
| Participant_26 | UV | M | Traumatic | L | 12 | 2023 | 0 | no | yes | no |
| Participant_27 | UV | F | Schanomma | L | 66 | NA | 120 | no | yes | yes |
| Participant_28 | UV | M | Traumatic | R | 11 | 2018 | 3 | no | yes | no |
| Participant_29 | UV | M | Schanomma | L | 34 | 2014 | 3 | no | yes | yes |
| Participant_30 | UV | M | Idiopathic | R | 8 | 2018 | 0 | no | yes | yes |
| Participant_31 | UV | F | Schanomma | L | 46 | NA | 3 | yes | yes | yes |
| Participant_32 | UV | M | Schanomma | R | 28 | 2021 | 0 | yes | yes | yes |
| Participant_33 | UV | F | Idiopathic | R | 2 | 2018 | NA | yes | yes | no |
| Participant_34 | UV | F | Schanomma | R | 20 | NA | NA | yes | no | NA |
| Participant_35 | UV | F | Arachnoid cyst | R | 42 | 2023 | 3 | yes | yes | yes |
| Participant_36 | UV | F | Auricular zoster | R | 10 | 2021 | 30 | no | yes | yes |
| Participant_37 | UV | M | Schanomma | R | 46 | 2021 | 6 | yes | yes | yes |
| Participant_38 | UV | M | Meniere | L | 20 | 2009 | 1 | yes | yes | yes |
| Participant_39 | UV | F | Idiopathic | R | 38 | 2016 | 72 | yes | yes | yes |
| Participant_40 | HS | F | NR | NR | NR | NR | NR | NR | yes | |
| Participant_41 | HS | M | NR | NR | NR | NR | NR | NR | yes | |
| Participant_42 | HS | M | NR | NR | NR | NR | NR | NR | yes | |
| Participant_43 | HS | F | NR | NR | NR | NR | NR | NR | yes | |
| Participant_44 | HS | M | NR | NR | NR | NR | NR | NR | yes | |
| Participant_45 | HS | M | NR | NR | NR | NR | NR | NR | no | |
| Participant_46 | HS | M | NR | NR | NR | NR | NR | NR | no | |
| Participant_47 | HS | F | NR | NR | NR | NR | NR | NR | yes | |
| Participant_48 | HS | F | NR | NR | NR | NR | NR | NR | yes | |
| Participant_49 | HS | M | NR | NR | NR | NR | NR | NR | yes | |
| Participant_50 | HS | F | NR | NR | NR | NR | NR | NR | yes | |
| Participant_51 | HS | M | NR | NR | NR | NR | NR | NR | yes | |
| Participant_52 | HS | F | NR | NR | NR | NR | NR | NR | no | |
| Participant_53 | HS | F | NR | NR | NR | NR | NR | NR | yes | |
| Participant_54 | HS | F | NR | NR | NR | NR | NR | NR | yes | |
| Participant_55 | HS | M | NR | NR | NR | NR | NR | NR | yes | |
| Participant_56 | HS | F | NR | NR | NR | NR | NR | NR | yes | |
| Participant_57 | HS | M | NR | NR | NR | NR | NR | NR | yes | |
| Participant_58 | HS | F | NR | NR | NR | NR | NR | NR | yes | |
| Participant_59 | HS | M | NR | NR | NR | NR | NR | NR | yes | |

Supplementary Table 1. Population characteristics regarding the pathology. DHI: Dizziness handicap inventory questionnaire; BV: Bilateral vestibulopathy; UV: Unilateral vestibulopathy; F: Female; M: Male; NA: Not answered; NR: Not relevant; For diagnostic delay, 0 means that the diagnostic was made a few hours after the onset of the first symptoms.
